# Estimates of Overall Survival in Patients With Cancer Receiving Different Treatment Regimens: Emulating Hypothetical Target Trials in the Surveillance, Epidemiology, and End Results (SEER)–Medicare Linked Database

**DOI:** 10.1001/jamanetworkopen.2020.0452

**Published:** 2020-03-05

**Authors:** Lucia C. Petito, Xabier García-Albéniz, Roger W. Logan, Nadia Howlader, Angela B. Mariotto, Issa J. Dahabreh, Miguel A. Hernán

**Affiliations:** 1 Department of Epidemiology, Harvard T.H. Chan School of Public Health, Boston, Massachusetts; 2 Now with the Feinberg School of Medicine, Division of Biostatistics, Department of Preventive Medicine, Northwestern University, Chicago, Illinois; 3 Mongan Institute, Massachusetts General Hospital, Boston; 4 RTI Health Solutions, Barcelona, Spain; 5 Division of Cancer Control and Population Sciences, National Cancer Institute, Bethesda, Maryland; 6 Center for Evidence Synthesis in Health, Brown University, Providence, Rhode Island; 7 Department of Health Services, Policy and Practice, Brown University, Providence, Rhode Island; 8 Department of Epidemiology, Brown University, Providence, Rhode Island; 9 Department of Biostatistics, Harvard T.H. Chan School of Public Health, Boston, Massachusetts; 10 Harvard-MIT Health Sciences and Technology, Harvard Medical School, Boston, Massachusetts

## Abstract

**Importance:**

The Surveillance, Epidemiology, and End Results (SEER)–Medicare linked database may provide insights into the comparative effectiveness of oncological treatments for elderly individuals who are underrepresented in clinical trials.

**Objective:**

To evaluate the suitability of SEER-Medicare data for assessing the effectiveness of adding a drug to an existing treatment regimen on the overall survival of elderly patients with cancer.

**Design, Setting, and Participants:**

This comparative effectiveness study analyzed SEER-Medicare data from 9549 individuals who received a new diagnosis of stage II colorectal cancer (2008-2012) and 940 patients who received a new diagnosis of advanced pancreatic adenocarcinoma (2007-2012), with follow-up to December 31, 2013 (SEER-Medicare data released in 2015). Two (hypothetical) target trials were designed and emulated based on 2 existing randomized clinical trials: (1) adjuvant fluorouracil after curative surgery for individuals with stage II colorectal cancer and (2) erlotinib added to gemcitabine for individuals with advanced pancreatic adenocarcinoma. Data were analyzed January 2018 to March 2019.

**Exposures:**

The following treatment strategies were compared: (1) fluorouracil initiation vs no initiation within 3 months of tumor resection and (2) erlotinib initiation vs no initiation within 12 weeks of gemcitabine initiation.

**Main Outcomes and Measures:**

All-cause mortality within 60 months of baseline for the fluorouracil trial and within 72 weeks for the erlotinib trial.

**Results:**

Compared with 3293 individuals in the existing fluorouracil trial, 9549 eligible individuals included in the present analyses were more likely to have colon cancer (8565 [90%] vs 2291 [71%]) and were older (median [interquartile range], 79 [73-84] vs 63 [56-68] years). The 5-year risk difference for initiation vs noninitiation of fluorouracil after surgery was −3.8% (95% CI, −14.8% to 12.6%), and the mortality hazard ratio (HR) was 0.95 (95% CI, 0.85-1.04). Compared with 569 individuals in the existing erlotinib trial, 940 eligible patients included in the present analysis were older (median [range], 74 [66-93] vs 64 [36-92] years) and more likely to be male (547 [58%] vs 298 [52%]). The 1-year risk difference for initiation vs noninitiation of erlotinib was 4.7% (95% CI, −9.4% to 18.0%), and the corresponding mortality HR was 1.04 (95% CI, 0.86-1.42). In naive analyses, the mortality HR estimate was 1.14 (95% CI, 0.95-1.36) for the fluorouracil emulation and 0.68 (95% CI, 0.54-0.87) for the erlotinib emulation.

**Conclusions and Relevance:**

The present estimates were similar to those from randomized clinical trials that studied adding the same cancer drugs to existing regimens. The published HR was 1.02 (95% CI, 0.70-1.48) in the fluorouracil trial for individuals aged 70 or older and 0.96 (95% CI, 0.74-1.24) in the erlotinib trial for individuals aged 65 years or older. The SEER-Medicare database may be adequate for studying the real-world effectiveness of adding a drug to treatment regimens used for elderly individuals with cancer.

## Key Points

**Question:**

Does the Surveillance, Epidemiology, and End Results–Medicare linked database contain sufficient information to estimate the effectiveness of adding a drug to an existing treatment regimen for elderly individuals with cancer who are systematically underrepresented in randomized trials?

**Findings:**

After explicitly emulating target trials, findings from this comparative effectiveness study using Surveillance, Epidemiology, and End Results–Medicare data were not meaningfully different from those in elderly subgroup analyses reported from randomized trials. Naive observational estimates, however, were not compatible with those from previous trials.

**Meaning:**

Analyses using Surveillance, Epidemiology, and End Results–Medicare data may be informative for some research questions examining the comparative effectiveness of oncological therapies for elderly individuals.

## Teaser

This comparative effectiveness study examines the suitability of using SEER-Medicare linked data to assess the overall survival associated with adding a drug to an existing treatment regimen for elderly patients with cancer.

## Introduction

When randomized clinical trials are not available at the time a clinical decision needs to be made, clinicians often must choose between making decisions based on either observational data or no human data at all. A cautious use of observational data has been recently endorsed by the American Society of Clinical Oncology, which states that observational studies can “inform questions that either have not been or cannot be answered by randomized trials”^1^^(p1845)^ and “complement the evidence collected in randomized trials.”^1^^(p1845)^ Observational studies may be particularly important for patients who are underrepresented in trials, such as elderly patients.^2,3^

Although health care databases make it possible to study cancer treatments as they are used in clinical practice, observational analyses are subject to several sources of bias.^4^ Some of these biases, however, can be eliminated by using observational data to emulate a (hypothetical) pragmatic target trial.^5,6^ Explicitly emulating a target trial is important because some well-known failures of observational research^7,8,9^ were the result of deviating from basic principles of study design rather than with the shortcomings inherent to observational data.

Here, we demonstrate how to use the Surveillance, Epidemiology, and End Results (SEER)–Medicare database^10^—a linkage between the SEER cancer registries and Medicare—to emulate target trials that compare cancer treatments for elderly individuals. Our goal is to describe procedures to increase the validity of comparative effectiveness analyses using the SEER-Medicare database.^11,12,13^ To demonstrate these procedures, we emulated 2 target trials for estimating the effect of the addition of a cancer drug to an existing treatment regimen on overall survival: (1) adjuvant fluorouracil after curative surgery in individuals with stage II colorectal cancer and (2) the addition of erlotinib to a regimen of gemcitabine for individuals with advanced pancreatic adenocarcinoma. As a benchmark, we used the age-specific estimates from 2 published randomized clinical trials,^14,15^ both of which estimated a near-null and imprecise effect for elderly individuals: hazard ratios (HRs) of 1.02 (95% CI, 0.70-1.48) for fluorouracil and 0.96 (95% CI, 0.74-1.24) for erlotinib. We also compared the emulated randomized clinical trial estimates with those from naive observational analyses that did not attempt to emulate a target trial.

## Methods

### Study Data: SEER-Medicare Linked Database

The SEER-Medicare database is a linkage of patient demographic and cancer-related variables collected by 17 SEER cancer registries across 12 states with Medicare claim files from the Centers for Medicare & Medicaid Services. The SEER data are summarized in the Patient Entitlement and Diagnosis Summary File, which is linked to Medicare claims. More information about the SEER-Medicare linked database can be found elsewhere.^16^ This study followed the Strengthening the Reporting of Observational Studies in Epidemiology (STROBE) reporting guideline and the International Society for Pharmacoeconomics and Outcomes Research (ISPOR) reporting guideline for good practices for comparative effectiveness research.^17,18^ The institutional review board at the Harvard T.H. Chan School of Public Health deemed this study exempt from review because the research involves the study of publicly available deidentified data.

To identify eligible individuals, construct a combined comorbidity score^19^ and a performance status proxy score,^20,21^ and also identify all other variables in this study, we used Medicare claims from the Inpatient, Outpatient, Home Health Agency, Durable Medical Equipment, Medpar, National Claims History, and Patient Entitlement and Diagnosis Summary Files. To identify treatment, we used the Healthcare Common Procedure Coding System and *Common Procedural Terminology* codes. Because erlotinib is an oral medication, we determined its use by records of filled prescriptions in Medicare Part D, which started in 2007. See eAppendix 1 in the Supplement for details on data use.

### Emulating a Target Trial in the SEER-Medicare Linked Database

Our approach had 2 steps. The first step was to fully articulate the clinical question by specifying the protocol of the (hypothetical) target trial.^5^ The second step was to emulate the target trial using the observational data. Table 1 and Table 2 outline the specifications of 2 target trials: (1) the addition of fluorouracil in stage II colorectal cancer and (2) the use of erlotinib in advanced pancreatic adenocarcinoma, based on existing randomized trials of fluorouracil^14^ and erlotinib^15^ (eTable 1 and eTable 2 in the Supplement). Table 1 and Table 2 also summarize the emulation procedure for both target trials using SEER-Medicare data. Key steps of the procedure are described herein.

**Table 1.** Protocol of the Target Trial to Study Adjuvant Fluorouracil-Based Chemotherapy in Stage II Colorectal Cancer and Emulation Procedure Using the SEER-Medicare Database

Protocol Component	Description of Target Trial	Description of Emulation Using SEER-Medicare Data
Eligibility criteria	Histologic diagnosis of stage II colorectal cancer (node negative) between 2008 and 2012 Aged into Medicare and was continuously enrolled in Parts A and B and not enrolled in an HMO for 12 mo before diagnosis Evidence of complete resection of colon or rectal cancer No history of cancer except nonmelanoma skin cancer No prior chemotherapy	Same as target trial
Treatment strategies^a^	Initiate any dose of fluorouracil as first line treatment up to 3 mo after postsurgery hospital discharge No chemotherapy initiated within 3 mo of postsurgery hospital discharge	Same as target trial
Assignment procedures	Participants were randomized to a treatment strategy at baseline and were aware of the assigned strategy	Randomization was assumed conditional on baseline covariates: year, sex, race/ethnicity, marital status, region of the United States, metropolitan county, median household income in census tract, percentage of households under poverty line in census tract, time between diagnosis and surgery, prolonged hospitalization after surgery, preoperative radiotherapy, cancer type (colon, rectum, or both), tumor grade, and in the year before surgery, anemia, abdominal distention, abnormal weight loss, asthenia, change in bowel movements, constipation, diarrhea, irritable bowel syndrome, No. of emergency department visits, colonoscopy, and abdominal or pelvic CT scan
Follow-up	For each eligible person, follow-up started when the person was assigned to a treatment strategy at postsurgery discharge from the hospital and ended at the earliest of death, loss of insurance eligibility, December 31, 2013, or 60 mo	Same as target trial
Outcome	Death certified by a physician, reported to Medicare, and confirmed by the National Death Index	Same as target trial
Causal contrast	Intention-to-treat effect; per-protocol effect	Per-protocol effect
Analysis plan	Intention-to-treat analysis; per-protocol analysis: inverse probability weighted pooled logistic regression model with censoring at deviation from protocol. Weights estimated as a function of baseline and postbaseline covariates: anemia, abdominal distention, abnormal weight loss, asthenia, change in bowel movements, constipation, diarrhea, irritable bowel syndrome, No. of emergency department visits, colonoscopy, and abdominal or pelvic CT scan	Same as target trial, except the per-protocol analysis was conducted in an expanded data set that included 2 replicates (1 per treatment strategy) of each eligible individual

Abbreviations: CT, computed tomography; HMO, health maintenance organization; SEER, Surveillance, Epidemiology, and End Results.

^a^ Under both strategies, the decision to discontinue fluorouracil at any time or start additional therapies after 3 months was left to the patient and physician’s discretion.

**Table 2.** Protocol of the Target Trial to Study the Addition of Erlotinib to a Regimen of Gemcitabine in Locally Advanced or Metastatic Pancreatic Cancer and Emulation Procedure Using the SEER-Medicare Database

Protocol Component	Description of Target Trial	Description of Emulation Using SEER-Medicare Data
Eligibility criteria	Histologic diagnosis of adenocarcinoma of the pancreas between April 2007 and July 2013	Same as target trial, except individuals with late-stage diagnoses could initiate gemcitabine beginning 2 wk before official confirmation of diagnosis
	Aged into Medicare, continuously enrolled in Parts A and B for 12 mo and Part D for 3 mo; and not enrolled in an HMO for 12 mo before diagnosis	
	No history of cancer except nonmelanoma skin cancer	
	If cancer stage IV or stage III with no surgery: Initiation of gemcitabine (any dose) within 12 wk of cancer diagnosis No prior chemotherapy or radiotherapy	
	If cancer stage I, II, or III with surgery (recurrence): Initiation of gemcitabine (any dose) 12 wk after surgery No chemotherapy or radiation after surgery	
Treatment strategies^a^	Initiated erlotinib (any dose) up to 12 wk after gemcitabine initiation Did not initiate erlotinib	Same as target trial
Assignment procedures	Participants were randomized to a treatment strategy at baseline and were aware of the assigned strategy	Randomization was assumed conditional on baseline covariates: tumor stage, age at diagnosis, and in the year before diagnosis, number of emergency department visits, Charlson Comorbidity index, performance status, cholangitis, and pneumonia
Follow-up	For each eligible person, follow-up started when a person is assigned to a treatment strategy and ended at the earliest of death, loss to insurance eligibility, December 31, 2013, or 18 mo	Same
Outcome	Death certified by a physician, reported to Medicare, and confirmed by the National Death Index	Same as target trial
Causal contrast	Intention-to-treat effect; per-protocol effect	Per-protocol effect only
Analysis plan	Intention-to-treat analysis; per-protocol analysis: inverse probability weighted pooled logistic regression model with censoring at deviation from protocol. Weights estimated as a function of baseline and postbaseline covariates: number of emergency department visits, Charlson Comorbidity Index, cholangitis, and pneumonia in previous week	Same, except the analysis was conducted in an expanded data set that included 2 replicates (1 per treatment strategy) of each eligible individual

Abbreviations: HMO, health maintenance organization; SEER, Surveillance, Epidemiology, and End Results.

^a^ Under both strategies, the decision to discontinue gemcitabine or erlotinib, as well as to initiate any additional therapies, was left to the patient and physician’s discretion.

#### Eligibility Criteria

The patient eligibility process is given in eFigure 1 and eFigure 2 in the Supplement. In addition to the eligibility criteria of the existing trials, our target trials also required having 1 year of continuous enrollment in Medicare Parts A and B, without being enrolled in a health maintenance organization, and having less than 3 months between diagnosis and initiation of first-line treatment.

For the fluorouracil trial, there were no eligibility restrictions related to the use of neoadjuvant chemotherapy or radiation. In addition, because some colorectal cancers are diagnosed during surgery, we allowed surgery to occur up to 4 weeks before the recorded date of cancer diagnosis to account for time spent in pathologic confirmation of the diagnosis.

For the erlotinib target trial, 3 months of enrollment in Medicare Part D was also required. In addition, individuals with an early cancer stage (I, II, or III) who had undergone surgery to remove the tumor began a second eligibility period if they initiated gemcitabine at least 12 weeks after surgery (otherwise gemcitabine was considered adjuvant care rather than treatment for progression) and before initiating other adjuvant chemotherapy or radiation; this modification emulated the randomized clinical trial conducted by Moore et al^15^ (eTable 2 in the Supplement).

#### Treatment Strategies and Assignment

The treatment strategies compared in our hypothetical target trials mimicked those in 2 trials: (1) initiation of fluorouracil vs no initiation of fluorouracil and (2) initiation of erlotinib vs no initiation of erlotinib in individuals receiving gemcitabine. All components of the treatment strategy needed to be in place within a prespecified period (the grace period) after baseline. Because the existing trials did not explicitly specify the length of the grace period, we chose a 3-month period based on our understanding of the clinical management of stage II colorectal cancer and of advanced pancreatic adenocarcinoma.

Observational analyses attempt to emulate randomized assignment via adjustment for confounders (ie, potential prognostic factors that are imbalanced between treatment groups at baseline). For example, performance status was expected to be a confounder for the effect of erlotinib, whereas perceived risk of relapse (summarized here by size of the tumor, grade of differentiation, number of resected nodes, and T category) was expected to be a confounder for that of fluorouracil. We adjusted for those factors and demographic characteristics, geographic characteristics, and comorbidities (as identified by previously validated procedure and diagnosis codes^20,21,22,23,24,25,26^) listed in Table 1 and Table 2. Target trials must be pragmatic trials in which treatment assignment is not blinded because observational data typically cannot be used to emulate double-blind trials.^5^

#### Start and End of Follow-up

The start of follow-up (baseline or time zero) for each individual was the time of first eligibility: the date of the first gemcitabine claim while otherwise eligible in the emulation of the erlotinib trial and the date of hospital discharge after surgery while otherwise eligible in the emulation of the fluorouracil trial. The end of follow-up was death, loss of insurance eligibility follow-up (loss of enrollment in Parts A, B, or D; enrollment in a health maintenance organization), or administrative end of follow-up (December 31, 2013; 60 months for the fluorouracil trial and 72 weeks for the erlotinib trial), whichever was earliest.

#### Outcome

The primary outcome of interest in both target trials was all-cause mortality. The SEER-Medicare database contains information about the date of death in both the SEER file and the Medicare claims. We used the Medicare date of death, which is more up to date and is validated by the National Death Index.^27^ The SEER-Medicare data do not include enough information to reliably study progression-free survival.^28^

#### Causal Contrast

Our causal contrast of interest was the per-protocol effect, that is, the effect that would have been observed if all trial participants had adhered to the treatment strategies specified in the protocol.^29^ Because our treatment strategies simply required the initiation of either fluorouracil or erlotinib within the grace period (regardless of future continuation), our causal contrast was similar to the intention-to-treat effect in a trial in which all individuals initiated their assigned treatment during the grace period, even if some of them later discontinued treatment.

### Statistical Analysis

We refer to analyses whose goal was to estimate the per-protocol effect as “per-protocol analyses”; these analyses should not to be confused with naive per-protocol analyses conducted in some trials.^5^ There are 3 steps of the per-protocol analysis that we implemented in our emulation of the target trials, which could also be implemented in target trials if they were actually conducted. (Technical details are provided in eAppendix 2 in the Supplement.)

For the first step, we censored individuals if or when they deviated from their assigned protocol: individuals who (1) initiated fluorouracil in the observation strategy or (2) initiated erlotinib in the gemcitabine alone strategy. In addition, because eligible individuals could not be uniquely assigned to a strategy at baseline when emulating target trials with a grace period, we duplicated (“cloned”) eligible individuals and assigned 1 clone to each treatment strategy (eFigure 3 in the Supplement).^30^ We then censored clones if they had no data compatible with their assigned treatment strategy by the end of the grace period.^6,30^ Details on all possible censoring schemes are given in eAppendix 2 in the Supplement.

Second, we used inverse probability weighting to adjust for the potential selection bias introduced by censoring.^31^ To do so, we estimated stabilized inverse probability weights as a function of the variables listed in Table 1 and Table 2.^32,33^ Third, we fit an inverse probability weighted discrete-time hazard model^34,35^ by pooled logistic regression, with death as the response and the following regressors: the indicator for the assigned treatment strategy, a function of time of follow-up (restricted cubic spline with knots at 3, 16, 30, 44, and 57 months for the fluorouracil emulation; quadratic for the erlotinib emulation), product terms for treatment strategy and time, and baseline covariates. To obtain standardized, treatment-specific risks for time points between baseline and the end of follow-up, we standardized the model-derived estimated values to the joint distribution of the baseline covariates. To estimate the mean HR commonly reported in randomized clinical trials, we fit a Cox proportional hazards model with treatment strategy as the only covariate, as described elsewhere.^36^ The final models are described in eAppendix 3 and eAppendix 4 in the Supplement.

We conducted several sensitivity analyses to test the robustness of our estimates to different choices of functional forms and grace periods (results were similar; see eAppendix 5 and eAppendix 6 in the Supplement). We computed 95% CIs via a nonparametric bootstrap based on 500 resamplings. We used SAS, version 9.4 (SAS Institute Inc), for data processing, and R, version 3.4.4 (The R Foundation), for data analyses. The SEER-Medicare data were from those released in 2015 (containing content through December 31, 2013), and our analyses were conducted from January 2018 to March 2019.

## Results

We identified 9549 eligible patients for the fluorouracil trial and 940 for the erlotinib trial, as described in eFigure 1 and eFigure 2 in the Supplement, respectively.

### Fluorouracil for Stage II Colorectal Cancer

The baseline characteristics of the eligible individuals are given eTable 3 in the Supplement. Compared with 3293 individuals in the existing randomized clinical trial,^14^ 9549 eligible individuals included in the present analyses were more likely to have colon cancer (8565 [90%] vs 2291 [71%]) and were older (median [interquartile range] age, 79 [73-84] years vs 63 [56-68] years) (eTable 4 in the Supplement).

Of the 9549 eligible individuals included in the present analysis (23 447 person-years of follow-up), 204 initiated fluorouracil-based chemotherapy within 3 months of their hospital discharge. Fluorouracil initiation was more likely for people who were younger, married, and had a T4 tumor category at diagnosis and rectum involvement (eAppendix 3 in the Supplement). Individuals were less likely to initiate fluorouracil-based chemotherapy if they had a prolonged hospitalization after surgery, preoperative radiotherapy, and, in the year before diagnosis, anemia and asthenia. By the end of the grace period, 185 individuals remained in the fluorouracil group and 6150 in the observation group (eTable 3 in the Supplement).

**Figure 1.**
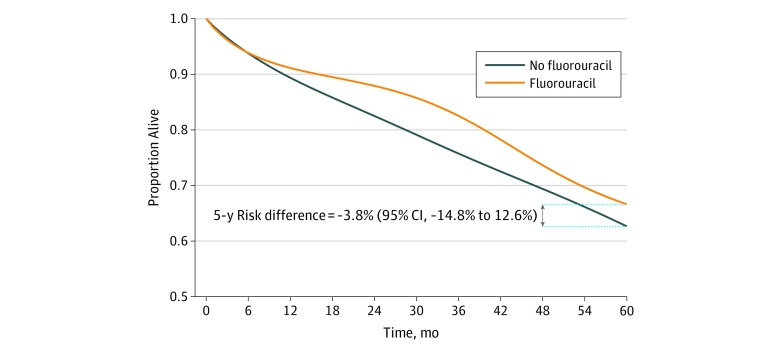
Survival Curves From the Emulated Target Trial of Adjuvant Fluorouracil-Based Chemotherapy in Stage II Colorectal Cancer Using Surveillance, Epidemiology, and End Results–Medicare Data From 2008 to 2013

There were 2148 deaths during follow-up. The adjusted estimated 5-year survival was 66.6% for fluorouracil and 62.7% for no fluorouracil; the 5-year risk difference was −3.8% (95% CI, −14.8% to 12.6%) (Figure 1). The mortality HR for fluorouracil vs no fluorouracil was 1.00 (95% CI, 0.89-1.12) without any adjustment, 1.01 (95% CI, 0.97-1.05) after adjustment for baseline covariates, and 0.95 (95% CI, 0.85-1.04) after adjustment for baseline and postbaseline covariates.

### Erlotinib for Advanced Pancreatic Cancer

The baseline characteristics of the eligible individuals are given in eTable 5 in the Supplement. Compared with 569 individuals in the existing randomized clinical trial,^15^ 940 individuals included in the present analysis were older (median [range] age, 74 [66-93] years vs 64 [36-92] years), more likely to be male (547 [58%] vs 298 [52%]), and less likely to have undergone prior therapy (radiotherapy: 9 [1%] vs 47 [8%]; chemotherapy: 53 [6%] vs 45 [8%]). In addition, all participants in the existing trial had a good performance status at enrollment, whereas 82 of the eligible individuals in our study (9%) had a poor performance status^20,21^ (eTable 6 in the Supplement).

Of the 940 eligible individuals (412 person-years of follow-up), 62 initiated erlotinib within 12 weeks of their initial gemcitabine dose. Erlotinib initiation was more likely for individuals with stage IV cancer and for those with comorbidities, and less likely for those with a poor performance status and older age (eAppendix 4 in the Supplement). By the end of the grace period, 44 individuals remained with the gemcitabine plus erlotinib strategy and 480 remained with the gemcitabine alone strategy (eTable 5 in the Supplement).

**Figure 2.**
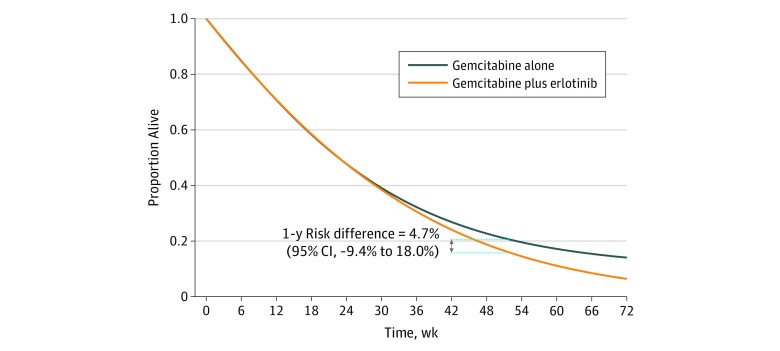
Survival Curves From the Emulated Target Trial of the Addition of Erlotinib to a Regimen of Gemcitabine in Locally Advanced or Metastatic Pancreatic Cancer Using Surveillance, Epidemiology, and End Results–Medicare Data From 2007 to 2013

There were 659 deaths during follow-up. The adjusted estimated 1-year survival was 15.6% for gemcitabine plus erlotinib and 20.4% in gemcitabine alone; the risk difference was 4.7% (95% CI, −9.4% to 18.0%) (Figure 2). The all-cause mortality HR for gemcitabine plus erlotinib vs gemcitabine alone was 1.08 (95% CI, 0.93-1.25) without any adjustment, 1.03 (95% CI, 0.96-1.09) after adjustment for baseline covariates, and 1.04 (95% CI, 0.86-1.42) after adjustment for baseline and postbaseline covariates.

### Naive Analyses

For comparison purposes, we conducted an analysis without cloning, censoring by deviation from protocol, or inverse probability weighting. Eligible individuals for the fluorouracil comparison were assigned to the fluorouracil group if they started fluorouracil therapy at any time during the follow-up and to the no fluorouracil group otherwise. We then fit a Cox proportional hazards regression model with treatment group and the baseline covariates. The mortality HR estimate was 1.14 (95% CI, 0.95-1.36). In a similar analysis for the erlotinib comparison, the mortality HR estimate was 0.68 (95% CI, 0.54-0.87).

## Discussion

We described 2 target trials for the addition of a cancer drug to an existing treatment regimen. These target trials were based on 2 existing randomized clinical trials: 1 pragmatic trial (fluorouracil) and 1 placebo-controlled, double-blind trial (erlotinib).^14,15^ We emulated these target trials using observational data from individuals 65 years of age or older whose data were in the SEER-Medicare database. Our observational estimates were consistent with the null estimates reported by the existing trials when we explicitly emulated the target trials but not when we conducted naive analyses that did not attempt to emulate a target trial.

The first emulation yielded a mortality HR of 0.95 (95% CI, 0.85-1.04) for fluorouracil vs no fluorouracil initiation, which is compatible with that reported in the actual fluorouracil trial^14^ for individuals 70 years of age or older (HR, 1.02; 95% CI, 0.70-1.48). Five-year survival could not be directly compared because the actual trial did not report these numbers for the elderly individuals. The second emulation yielded a mortality HR of 1.04 (95% CI, 0.86-1.42) for erlotinib vs no erlotinib initiation after gemcitabine, which is compatible with that reported in the existing erlotinib trial^15^ for individuals 65 years of age or older (HR, 0.96; 95% CI, 0.74-1.24). The estimated 1-year survival in the erlotinib strategy was approximately 16% in the emulated trial and 23% in the existing trial; the estimated 1-year survival in the gemcitabine alone strategy was approximately 20% in the emulated trial and 17% in the existing trial.

The target trial framework requires data sources with adequate longitudinal information on treatments, outcomes, and confounders. We were able to obtain that information from the linkage of detailed cancer information from the SEER registries with administrative data from Medicare claims. Detailed timing information for comorbidity onset and treatment administration exists solely in Medicare; thus, using SEER only would preclude us from emulating target trials. The use of SEER registry data alone may have contributed to erroneous conclusions in recent reports (later retracted)^37,38^ that compared cancer treatments in the elderly population. The use of Medicare data enabled us to reduce some errors present in SEER alone (eg, in the radiation variable) but does not guarantee perfect coding reliability of all variables (eg, rare histologic results).^39^

Similar to all observational analyses, ours assume that the lack of randomized treatment assignment can be approximately replaced by adjustment for the measured confounders. In our analyses, after the target trial was specified and the emulation procedures followed, we found no indication of strong confounding by measured variables. In fact, adjustment for multiple prognostic factors only modestly changed the estimates in both emulations. Given that we adjusted for some of the most important indications for the studied treatments, it is likely that confounding was not the most important validity threat in our examples.

Leaving aside a lack of randomization, a major threat to the validity of observational comparative effectiveness analyses is a failure to emulate components of a target trial other than randomization, which may introduce biases such as selection bias and immortal time bias. Explicit emulation of a target trial helps eliminate these problems.^6,23^

Similar to our naive analyses, previous uses of SEER-Medicare data to estimate treatment effects that did not explicitly attempt to emulate a target trial may have introduced immortal time bias.^40^ For the same reasons, prior comparisons of randomized clinical trials and observational studies based on SEER-Medicare^41^ or other databases^42^ are difficult to interpret because, in the absence of an explicit emulation of a target trial, any discrepancies may be due to incorrect analysis rather than to inherent limitations of the observational data.

### Limitations

The use of large observational databases does not guarantee transportable or precise estimates. The requirements of target trial emulation (eg, requiring individuals to participate in Medicare Part D, which was necessary in the erlotinib analysis) may limit the generalizability of our results, although probably not as much as do the eligibility criteria of actual randomized clinical trials. Perhaps the most surprising aspect of our analyses was the small sample sizes despite the apparently large amount of data in the SEER-Medicare database. Because few eligible individuals received the adjuvant therapy of interest, there were fewer than 200 individuals in the fluorouracil strategy after 3 months of follow-up and fewer than 190 individuals in the erlotinib strategy after 12 weeks of follow-up. When, as in our analyses, few individuals follow the treatment strategies of interest, the researchers’ ability to adequately control for confounding and selection bias is limited because not many covariates can be added to the regression models.

## Conclusions

We emulated 2 target trials for the addition of a cancer treatment by using the SEER-Medicare database. Our findings showed that, in some settings, observational estimates from this database are consistent with those from randomized clinical trials in elderly populations, but only when an appropriate causal inference approach is implemented.

As the US population ages, understanding how cancer treatments affect elderly individuals will have profound implications for clinical decision-making. Because elderly individuals tend to be underrepresented in randomized clinical trials,^2,3^ observational health care databases can be used to obtain more precise estimates in that segment of the population or, at the very least, to guide the design of future randomized clinical trials by helping to rule out clearly ineffective or harmful treatment strategies.

## Notes

Supplement. **eTable 1.** Protocol of the Target Trial to Study Adjuvant Fluorouracil-Based Chemotherapy in Stage II Colorectal Cancer and Protocol of the Existing QUASAR Trial (2007) **eTable 2.** Protocol of the Target Trial to Study the Addition of Erlotinib to a Regimen of Gemcitabine in Locally Advanced or Metastatic Pancreatic Cancer and Protocol of the Existing Trial (Moore et al. 2007) **eTable 3.** Characteristics of Eligible Individuals With Stage II Colorectal Cancer Who Were Included in the Emulation of the Fluorouracil Target Trial at Baseline and the End of the Grace Period (3 Months Post-Baseline), SEER-Medicare 2008-2013 **eTable 4.** Comparison of Individuals in the Existing QUASAR Trial (2007) in the Emulation of the Fluorouracil Target Trial Using SEER-Medicare 2008-2013 **eTable 5.** Characteristics of Eligible Individuals With Locally Advanced or Metastatic Pancreatic Cancer Who Were Included in the Emulation of the Erlotinib Target Trial at Baseline and the End of the Grace Period (12 Weeks Post-Baseline), SEER-Medicare 2007-2013 **eTable 6.** Comparison of Individuals in the Existing Trial (Moore et al. 2007) and in the Emulation of the Erlotinib Target Trial Using SEER-Medicare 2007-2013 **eFigure 1.** Flowchart of Eligibility for a Target Trial of Adjuvant Fluorouracil-Based Chemotherapy in Individuals With Stage II Colorectal Cancer, SEER-Medicare 2008-2013 **eFigure 2.** Flowchart of Eligibility for a Target Trial of Addition of Erlotinib to Gemcitabine in Individuals With Locally Advanced or Metastatic Pancreatic Cancer, SEER-Medicare 2007-2013 **eFigure 3.** Illustration of the Cloning and Censoring Process for the Fluorouracil Target Trial Emulation **eAppendix 1.** Codes Used to Identify Variables Used in the Analyses **eAppendix 2.** Details of Statistical Analysis **eAppendix 3.** Models Used in the Emulation of the Fluorouracil Target Trial **eAppendix 4.** Models Used in the Emulation of the Erlotinib Target Trial **eAppendix 5.** Sensitivity Analyses for the Fluorouracil Target Trial Emulation **eAppendix 6.** Sensitivity Analyses for the Erlotinib Target Trial Emulation
