# Errors in Results and Supplement

**DOI:** 10.1001/jamanetworkopen.2020.4966

**Published:** 2020-04-06

In the Original Investigation titled “Estimates of Overall Survival in Patients With Cancer Receiving Different Treatment Regimens: Emulating Hypothetical Target Trials in the Surveillance, Epidemiology, and End Results (SEER)–Medicare Linked Database,”^1^ published March 5, 2020, the authors were in violation of the SEER-Medicare data use agreement and had to remove data from 1 sentence in the Results and remove or replace data in the Supplement. The second sentence of the fifth paragraph of the Results should read as follows: “Compared with 569 individuals in the existing randomized clinical trial,^15^ 940 individuals included in the present analysis were older (median [range] age, 74 [66-93] years vs 64 [36-92] years), more likely to be male (547 [58%] vs 298 [52%]), and less likely to have undergone prior chemotherapy (53 [6%] vs 45 [8%]).” This article has been corrected.^1^
