# Supplementary material for: Estimates of Overall Survival in Patients With Cancer Receiving Different Treatment Regimens: Emulating Hypothetical Target Trials in the Surveillance, Epidemiology, and End Results (SEER)–Medicare Linked Database
Source: JAMA Netw Open. 2020 Mar 5;3(3):e200452. doi: 10.1001/jamanetworkopen.2020.0452 (PMC7059023; doi:10.1001/jamanetworkopen.2020.0452)
Supplement: Supplement. — eTable 1. Protocol of the Target Trial to Study Adjuvant Fluorouracil-Based Chemotherapy in Stage II Colorectal Cancer and Protocol of the Existing QUASAR Trial (2007) eTable 2. Protocol of the Target Trial to Study the Addition of Erlotinib to a Regimen of Gemcitabine in Locally Advanced or Metastatic Pancreatic Cancer and Protocol of the Existing Trial (Moore et al. 2007) eTable 3. Characteristics of Eligible Individuals With Stage II Colorectal Cancer Who Were Included in the Emulation of the Fluorouracil Target Trial at Baseline and the End of the Grace Period (3 Months Post-Baseline), SEER-Medicare 2008-2013 eTable 4. Comparison of Individuals in the Existing QUASAR Trial (2007) in the Emulation of the Fluorouracil Target Trial Using SEER-Medicare 2008-2013 eTable 5. Characteristics of Eligible Individuals With Locally Advanced or Metastatic Pancreatic Cancer Who Were Included in the Emulation of the Erlotinib Target Trial at Baseline and the End of the Grace Period (12 Weeks Post-Baseline), SEER-Medicare 2007-2013 eTable 6. Comparison of Individuals in the Existing Trial (Moore et al. 2007) and in the Emulation of the Erlotinib Target Trial Using SEER-Medicare 2007-2013 eFigure 1. Flowchart of Eligibility for a Target Trial of Adjuvant Fluorouracil-Based Chemotherapy in Individuals With Stage II Colorectal Cancer, SEER-Medicare 2008-2013 eFigure 2. Flowchart of Eligibility for a Target Trial of Addition of Erlotinib to Gemcitabine in Individuals With Locally Advanced or Metastatic Pancreatic Cancer, SEER-Medicare 2007-2013 eFigure 3. Illustration of the Cloning and Censoring Process for the Fluorouracil Target Trial Emulation eAppendix 1. Codes Used to Identify Variables Used in the Analyses eAppendix 2. Details of Statistical Analysis eAppendix 3. Models Used in the Emulation of the Fluorouracil Target Trial eAppendix 4. Models Used in the Emulation of the Erlotinib Target Trial eAppendix 5. Sensitivity Analyses for the Fluorouracil Target Trial Emulation eAp [file jamanetwopen-3-e200452-s001.pdf]

Supplementary Online Content

Petito LC, García-Albéniz X, Logan RW, et al. Estimates of overall survival in patients with cancer receiving different treatment regimens: emulating hypothetical target trials in the Surveillance, Epidemiology, and End Results (SEER)–Medicare linked database. *JAMA Netw Open*. 2020;3(3):e200452. doi:10.1001/jamanetworkopen.2020.0452

eTable 1. Protocol of the Target Trial to Study Adjuvant Fluorouracil-Based Chemotherapy in Stage II Colorectal Cancer and Protocol of the Existing QUASAR Trial (2007)

eTable 2. Protocol of the Target Trial to Study the Addition of Erlotinib to a Regimen of Gemcitabine in Locally Advanced or Metastatic Pancreatic Cancer and Protocol of the Existing Trial (Moore et al. 2007)

eTable 3. Characteristics of Eligible Individuals With Stage II Colorectal Cancer Who Were Included in the Emulation of the Fluorouracil Target Trial at Baseline and the End of the Grace Period (3 Months Post-Baseline), SEER-Medicare 2008-2013

eTable 4. Comparison of Individuals in the Existing QUASAR Trial (2007) in the Emulation of the Fluorouracil Target Trial Using SEER-Medicare 2008-2013

eTable 5. Characteristics of Eligible Individuals With Locally Advanced or Metastatic Pancreatic Cancer Who Were Included in the Emulation of the Erlotinib Target Trial at Baseline and the End of the Grace Period (12 Weeks Post-Baseline), SEER-Medicare 2007-2013

eTable 6. Comparison of Individuals in the Existing Trial (Moore et al. 2007) and in the Emulation of the Erlotinib Target Trial Using SEER-Medicare 2007-2013

eFigure 1. Flowchart of Eligibility for a Target Trial of Adjuvant Fluorouracil-Based Chemotherapy in Individuals With Stage II Colorectal Cancer, SEER-Medicare 2008-2013

eFigure 2. Flowchart of Eligibility for a Target Trial of Addition of Erlotinib to Gemcitabine in Individuals With Locally Advanced or Metastatic Pancreatic Cancer, SEER-Medicare 2007-2013

eFigure 3. Illustration of the Cloning and Censoring Process for the Fluorouracil Target Trial Emulation

eAppendix 1. Codes Used to Identify Variables Used in the Analyses

eAppendix 2. Details of Statistical Analysis

eAppendix 3. Models Used in the Emulation of the Fluorouracil Target Trial

eAppendix 4. Models Used in the Emulation of the Erlotinib Target Trial

eAppendix 5. Sensitivity Analyses for the Fluorouracil Target Trial Emulation

eAppendix 6. Sensitivity Analyses for the Erlotinib Target Trial Emulation

This supplementary material has been provided by the authors to give readers additional information about their work.

© 2020 Petito LC et al. *JAMA Network Open*.

eTable 1. Protocol of the Target Trial to Study Adjuvant Fluorouracil-Based Chemotherapy in Stage II Colorectal Cancer and Protocol of the Existing QUASAR Trial (2007)

**Protocol Component**	**Description of target trial**	**Description of existing trial**
Eligibility Criteria	– Histologic diagnosis of stage II colorectal cancer (node negative) between January 1, 2008 and December 31, 2012<br>– Medicare beneficiaries ages 66 years or older<br>o To satisfy insurance and entitlement requirements, individuals must have aged into Medicare and been continuously enrolled in Parts A & B and not enrolled in an HMO for 12 months before diagnosis.<br>– Evidence of complete resection of colon or rectal cancer with “uncertain indication for chemotherapy”<br>– No history of prior cancer (except non-melanoma skin cancer)<br>– No prior chemotherapy	– Histologic diagnosis of colorectal cancer with no evidence of distant metastases between May 1994 and December 2003<br>– Evidence of complete resection of colorectal cancer with “uncertain indication for chemotherapy”<br>– No definite contraindications to any of the chemotherapy regimens (determined by clinician)<br>– Resection margins and peritoneal washings negative for malignant cells
Treatment Strategies	**A.** Initiate any dose of fluorouracil as first line treatment up to 3 months after post-surgery hospital discharge.<br>**B.** Do not initiate any chemotherapy within 3 months	**A.** 30 doses of fluorouracil (370mg/m2 intravenously), given either as six 5-day courses with 4 weeks between the start of the courses or as 30 once-

© 2020 Petito LC et al. *JAMA Network Open*.

	of post-surgery hospital discharge<br><br>Under both strategies, the decision to discontinue fluorouracil or initiate any additional therapies is left to the patient and physician's discretion.	weekly doses. Ideally this treatment begins within 6 weeks of surgery. Patients can take high-dose L-folinic acid (175 mg intravenously), low-dose L-folinic acid (25 mg intravenously), or levamisole (50 mg) at their discretion.<br><br>**B.** Observation – do not initiate any chemotherapy
Assignment Procedures	Participants are randomized to either treatment strategy at baseline, and are aware of the strategy they are assigned to.	Participants are randomized to a strategy by phone call to a central office. A "minimized" randomization procedure was used, ensuring balance with respect to age-group, site of cancer, stage, portal-vein infusion, preoperative radiotherapy, planned postoperative radiotherapy, and chemotherapy schedule (weekly versus not). Treatments were balanced within participating centers.
Follow-up Period	Time zero of follow-up is the first time an individual meets all eligibility criteria (when the person is assigned to one of the treatment strategies), here assumed to be the date of post-surgery discharge from the hospital.<br><br>Follow-up ends at the earliest of death, loss to follow-up (loss of enrollment in Medicare Parts A or B; enrollment in an HMO), or administrative end of follow-up (December 31, 2013 or 60 months after time zero)	Follow-up begins at randomization.<br><br>Follow-up ends at the earliest of death, loss to follow-up, or administrative end of follow-up (January 2005 or 10 years after time zero).

© 2020 Petito LC et al. *JAMA Network Open*.

Outcome	All-cause mortality. Death certified by a physician, reported to Medicare and confirmed by the National Death Index within 5 years of time zero.	All-cause mortality within 10 years of time zero.
Causal contrasts of interest	Intention-to-treat effect: effect of being assigned to the strategies at baseline, regardless of whether individuals adhere to them during follow-up<br><br>Per-protocol effect: effect of adhering to the strategies (as defined in the protocol) during follow-up	Intention-to-treat effect only.
Analysis Plan	Intention-to-treat effect estimated via comparison of 5-year risk of all-cause mortality among individuals assigned to each treatment strategy from a pooled logistic regression model adjusted for baseline covariates.<br><br>Per-protocol effect estimates are calculated from an inverse probability weighted pooled logistic regression model, adjusted for baseline and post-baseline covariates: anemia, abdominal distention, abnormal weight loss, asthenia, change in bowel movements, constipation, diarrhea, irritable bowel syndrome, # of emergency department visits, colonoscopy, and abdominal or pelvic CT scan.	Intention-to-treat effect estimated via comparison of 10-year risk of all-cause mortality among individuals assigned to each treatment strategy using the Kaplan-Meier method.

© 2020 Petito LC et al. *JAMA Network Open*.

eTable 2. Protocol of the Target Trial to Study the Addition of Erlotinib to a Regimen of Gemcitabine in Locally Advanced or Metastatic Pancreatic Cancer and Protocol of the Existing Trial (Moore et al. 2007)

Protocol Component	Target trial	Description of existing trial
Eligibility Criteria	– Histologic diagnosis of adenocarcinoma of the pancreas between April 2007 and July 2013<br>– Medicare beneficiaries ages 66 years or older<br>o To satisfy insurance and entitlement requirements, individuals must have aged into Medicare and been continuously enrolled in:<br>▪ Parts A & B for 12 months before diagnosis<br>▪ Part D for 3 months before diagnosis<br>and not enrolled in an HMO for 12 months before diagnosis.<br>– No history of prior cancer (except non-melanoma skin cancer)<br>– If diagnosis at late stage (stage IV or stage III with no surgery):<br>o Initiation of gemcitabine (any dose) within 12 weeks of cancer diagnosis<br>o Treatment naïve<br>– If diagnosis at early stage (stage I, II, or III) with record of surgery (recurrence):<br>o Initiation of gemcitabine (any dose) after 12 weeks post-surgery<br>o No chemotherapy or radiation post-surgery	– Histologic or cytologic evidence of locally advanced or metastatic adenocarcinoma of the pancreas between October 2001 and January 2003<br>– ECOG performance status 0, 1, or 2<br>– Adequate hematologic, renal, and hepatic function<br>– No prior chemotherapy except fluorouracil or gemcitabine given concurrently as a radiosensitizer.

© 2020 Petito LC et al. *JAMA Network Open*.

Treatment Strategies	**A.** Initiate gemcitabine as first line treatment. Initiate erlotinib (any dose) within the **grace period:** up to 12 weeks after gemcitabine initiation.<br>**B.** Initiate gemcitabine as first line treatment within the grace period. Do not initiate erlotinib.<br><br>Under both strategies, the decision to discontinue gemcitabine or erlotinib, as well as to initiate any additional therapies, is left to the patient and physician's discretion.	**A.** Gemcitabine (1,000 mg/m^2 intravenously) plus erlotinib (100 or 150 mg/d orally)<br>**B.** Gemcitabine (1,000 mg/m^2 intravenously) plus placebo<br><br>Under both strategies, gemcitabine was administered on days 1, 8, 15, 22, 29, 36, and 43, followed by a 1-week rest, and on days 1, 8, and 15 in subsequent 4-week cycles. Erlotinib was taken once daily.
Assignment Procedures	Participants are randomized to either treatment strategy at baseline, and are aware of the strategy they are assigned to.	Patients are randomized to either treatment strategy at baseline, stratified by center, performance status (ECOG 0 versus 1-2), and stage (locally advanced versus metastatic). Patients and physicians are blinded to treatment assignment.
Follow-up Period	**Time zero** of follow-up is the first time an individual meets all eligibility criteria (when the person is assigned to one of the treatment strategies).<br>Follow-up ends at the earliest of death, loss to follow-up (loss of enrollment in Medicare Parts A, B, or D; enrollment in an HMO), or administrative end of follow-up (December 31, 2013 or 18 months after time zero)	Follow-up begins at randomization.<br>Follow-up ends at the earliest of death, loss to follow-up, or administrative end of follow-up (September 2004 or 24 months after time zero).
Outcome	All-cause mortality. Death certified by a physician, reported to Medicare and confirmed by the National Death Index within 18 months of time zero.	All-cause mortality within 24 months of baseline.
Causal contrasts of interest	Intention-to-treat effect: effect of being assigned to the strategies at baseline, regardless of whether individuals adhere to them during follow-up	Intention-to-treat effect only.

© 2020 Petito LC et al. *JAMA Network Open*.

	Per-protocol effect: effect of adhering to the strategies (as defined in the protocol) during follow-up	
Analysis Plan	Intention-to-treat effect estimated via comparison of 18-month risk of all-cause mortality among individuals assigned to each treatment strategy from a pooled logistic regression model adjusted for baseline covariates.<br><br>Per-protocol effect estimates are calculated from an inverse probability weighted pooled logistic regression model, adjusted for baseline and post-baseline covariates: number of emergency department visits, Charlson Comorbidity Index, cholangitis, and pneumonia (each defined using claims from the previous week).	Intention-to-treat effect estimated via comparison of 24-month risk of all-cause mortality among individuals assigned to each treatment strategy using the Kaplan-Meier method.

© 2020 Petito LC et al. *JAMA Network Open*.

eTable 3. Characteristics of Eligible Individuals With Stage II Colorectal Cancer Who Were Included in the Emulation of the Fluorouracil Target Trial at Baseline and the End of the Grace Period (3 Months Post-Baseline), SEER-Medicare 2008-2013

	**Baseline** Overall Sample	**3 months** No fluorouracil	**3 months** Fluorouracil
	N = 9,549	N = 6,150	N = 185
Demographics			
Sex			
Female	4,025 (42.2)	2,519 (41.0)	95 (48.6)
Male	5,524 (57.8)	3,631 (59.0)	90 (51.4)
Race			
Non-hispanic white	7,758 (81.2)	5,163 (84.0)	149 (80.5)
Non-hispanic black	676 (7.1)	392 (6.4)	15 (8.1)
Other	1,115 (11.7)	595 (9.7)	21 (11.4)
Age at diagnosis			
Median (IQR)	79 (73 to 84)	79 (74 to 85)	72 (68 to 76)
Year of DX			
2008-2009	5,002 (52.4)	3,291 (53.5)	103 (55.7)
2010-2011	4,547 (47.6)	2,859 (46.5)	82 (44.3)
Married	4,753 (49.8)	3,012 (49.0)	115 (62.2)
U.S. Region			
Midwest	1,191 (12.5)	862 (14.0)	34 (18.4)
Northeast	2,442 (25.6)	1,832 (29.8)	72 (38.9)
South	612 (6.4)	397 (6.5)	---
West	5,304 (55.5)	3,059 (49.7)	71 (38.4)
Urbanicity			
Non-metropolitan counties	3,049 (15.2)	1,061 (17.3)	45 (24.3)
Metropolitan counties	16,951 (84.8)	5,089 (82.7)	140 (75.7)
Census tract, Median (IQR)			
Household income in US dollars	51,084 (37,927 to 70,130)	50,445 (37,590 to 70,188)	45,903 (34,834 to 60,673)
Households below poverty, %	9.3 (5.0 to 16.3)	9.1 (4.9 to 15.8)	9.7 (5.5 to 17.9)
Highest household education, %			
No High school	24.1 (13.8 to 38.7)	24.2 (14.0 to 39.3)	20.7 (12.0 to 35.8)
High school	28.8 (23.3 to 34.6)	28.2 (22.9 to 34.0)	26.6 (21.4 to 32.9)
Some college	26.6 (19.1 to 34.3)	27.4 (19.6 to 35.1)	30.3 (22.9 to 38.6)
College or more	13.3 (7.4 to 22.5)	13.0 (7.3 to 21.9)	15.8 (8.4 to 25.2)
Tumor characteristics			
Site of tumor			
Colon (excluding appendix)	8,565 (89.7)	5,592 (90.9)	141 (76.2)
Rectum	456 (4.8)	235 (3.8)	26 (14.1)
Both	528 (5.5)	323 (5.3)	18 (9.7)
T4 tumor stage			
Yes	1,260 (13.2)	703 (11.4)	56 (30.3)

© 2020 Petito LC et al. *JAMA Network Open*.

No	8,289 (86.8)	5,447 (88.6)	129 (69.7)
Poor/undifferentiated tumor grade			
Yes	7,693 (80.6)	4,959 (80.6)	147 (79.5)
No	1,856 (19.4)	1,191 (19.4)	38 (20.5)
Surgery characteristics			
<12 lymph nodes examined at SX	1,732 (18.1)	1,031 (16.8)	48 (25.9)
Time from diagnosis to surgery			
-30 to 0 days	2,233 (23.4)	1,440 (23.4)	52 (28.1)
1 to 30 days	5,492 (57.5)	3,564 (58.0)	105 (56.8)
31 to 60 days	1,522 (15.9)	960 (15.6)	21 (11.4)
61 to 90 days	302 (3.2)	186 (3.0)	---
Hospitalization >14 days after surgery	978 (10.2)	586 (9.5)	13 (7.0)
Pre-operative radiotherapy	782 (8.2)	643 (10.5)	---
Healthcare Utilization in year before surgery			
Colonoscopy	5,506 (57.7)	4,797 (78.0)	128 (69.2)
Pelvic or Abdominal CT scan	5,744 (60.2)	4,917 (80.0)	150 (81.1)
At least one ER visit in			
year before diagnosis	4,692 (49.1)	453 (7.4)	---
Symptoms and Comorbidities			
Charlson Comorbidity Index			
Year before surgery, median (IQR)	1 (0 to 3)	2 (1 to 3)	1 (0 to 2)
Anemia	5,740 (60.1)	4,305 (70.0)	110 (59.5)
Abdominal distention	380 (4.0)	316 (5.1)	---
Abnormal weight loss	1,225 (12.8)	993 (16.1)	34 (18.4)
Asthenia	2,889 (30.3)	2,420 (39.3)	43 (23.2)
Change in bowel habit	903 (9.5)	750 (12.2)	31 (16.8)
Constipation	1,310 (13.7)	1070 (17.4)	35 (18.9)
Diarrhea	1,102 (11.5)	882 (14.3)	28 (15.1)
Irritable Bowel Syndrome	201 (2.1)	177 (2.9)	---

--- reported when cell size is ≤10, as per the SEER-Medicare Data Use Agreement

© 2020 Petito LC et al. *JAMA Network Open*.

eTable 4. Comparison of Individuals in the Existing QUASAR Trial (2007) in the Emulation of the Fluorouracil Target Trial Using SEER-Medicare 2008-2013

	SEER-Medicare Eligible Sample (n = 9,549)		QUASAR Participants (n = 3,239)	
	n	%	n	%
Site				
Colon	8,565	89.7	2291	70.7
Rectum (or both)	984	10.3	948	29.3
Sex				
Male	5,524	57.8	1979	61.1
Female	4,025	42.2	1260	38.9
Age				
<59	---	---	1225	37.8
60-69	1132	11.9	1351	41.7
70+	8417	88.1	663	20.5
Median age (IQR)	79		63	
IQR	73 to 84		56 to 68	
Other adjuvant therapy				
Pre-operative radiotherapy	782	8.2	203	6.3

IQR: Inner quartile range

--- reported when cell size is ≤10, as per the SEER-Medicare Data Use Agreement

eTable 5. Characteristics of Eligible Individuals With Locally Advanced or Metastatic Pancreatic Cancer Who Were Included in the Emulation of the Erlotinib Target Trial at Baseline and the End of the Grace Period (12 Weeks Post-Baseline), SEER-Medicare 2007-2013

	**Baseline** Overall Sample	**12 weeks** Gemcitabine Alone	**12 weeks** Gemcitabine + Erlotinib
	N = 940	N = 494	N = 44
Demographics			
Sex			
Female	393 (41.8)	196 (39.7)	19 (43.2)
Male	547 (58.2)	298 (60.3)	25 (56.8)
Race			
Non-hispanic white	772 (82.1)	401 (81.2)	34 (77.3)
Other	168 (17.9)	93 (18.8)	---
Age at diagnosis			
Median	74	74	73
Range	66-93	66-90	66-82
Year of diagnosis			
2008-2010	523 (55.6)	291 (58.9)	29 (65.9)
2011-2013	417 (44.4)	203 (41.1)	15 (34.1)
Married	535 (56.9)	274 (55.5)	(63.6)
U.S. Region			
Midwest	125 (13.3)	67 (13.6)	---
Northeast	275 (29.3)	154 (31.2)	---
South	161 (17.1)	83 (16.8)	---
West	379 (40.3)	190 (38.5)	29 (65.9)
Urbanicity			
Big Metro	552 (58.7)	289 (58.5)	21 (47.7)
Metro	223 (23.7)	121 (24.5)	16 (36.4)
Urban, less urban, rural	165 (17.6)	84 (17.0)	---
Tumor characteristics			
Tumor stage at diagnosis			
Ia	---	---	---
Ib	9 (1.0)	---	---
IIa	31 (3.3)	22 (4.5)	---
IIb	68 (7.2)	37 (7.5)	---
III	129 (13.7)	96 (19.4)	---
IV	700 (74.5)	332 (67.2)	38 (86.4)
Tumor grade at diagnosis			
1	33(3.5)	19 (3.8)	---
2	112 (11.9)	60 (12.1)	---
3	147 (15.6)	84 (17.0)	---
4	---	---	---
5+	641 (68.2)	329 (66.6)	31 (70.5)
Comorbidities (year prior to gemcitabine initiation)			

© 2020 Petito LC et al. *JAMA Network Open*.

Anemia	372 (39.6)	45 (9.1)	---
Cholangitis or biliary tract obstruction	348 (37.0)	11 (2.2)	---
Intestinal Obstruction	91 (9.7)	---	---
Performance status* (3+)	82 (8.7)	37 (7.5)	---
Pneumonia	85 (9.0)	---	---
Thrombolitic events (venous thrombosis, pulmonary embolism, acute myocardial infarction)	143 (15.2)	24 (4.9)	---
ER Visits (year prior to gemcitabine initiation)			
0	406 (43.2)		
1	272 (28.9)		
2	150 (16.0)		
3+	112 (11.9)		
Charlson Comorbidity Index (year prior to gemcitabine initiation)			
0	190 (20.2)		
1	264 (28.1)		
2	193 (20.5)		
3+	293 (31.2)		

* as defined in [18, 19]

--- reported when cell size is ≤10, as per the SEER-Medicare Data Use Agreement

© 2020 Petito LC et al. *JAMA Network Open*.

eTable 6. Comparison of Individuals in the Existing Trial (Moore et al. 2007) and in the Emulation of the Erlotinib Target Trial Using SEER-Medicare 2007-2013

	SEER-Medicare Eligible Sample		Moore et al. (2007) Participants	
	N = 940		N = 569	
	n	%	n	%
Sex				
Female	393	41.8	271	47.6
Male	547	58.2	298	52.4
Age, years				
Median	74.0		63.9	
Range	66.0-93.0		36.1-92.4	
ECOG performance status[a]				
0-2	858	91.3	569	100.0
3+	82	8.7	0	0.0
Extent of disease				
Locally advanced	240	25.5	138	24.3
Distant metastases	700	74.5	431	75.7
Prior therapy[b]				
Radiotherapy	---	---	47	8.3
Chemotherapy	53	5.6	45	7.9
Prior surgical resection of primary tumor	117	12.4	48	8.4

[a]ECOG performance status < 3 was an eligibility criteria for Moore et al. (2007)

[b]In SEER-Medicare eligible sample, prior therapy is only possible in individuals with prior surgical resection of primary tumor

--- reported when cell size is ≤10, as per the SEER-Medicare Data Use Agreement

© 2020 Petito LC et al. *JAMA Network Open*.

eFigure 1. Flowchart of Eligibility for a Target Trial of Adjuvant Fluorouracil-Based Chemotherapy in Individuals With Stage II Colorectal Cancer, SEER-Medicare 2008-2013

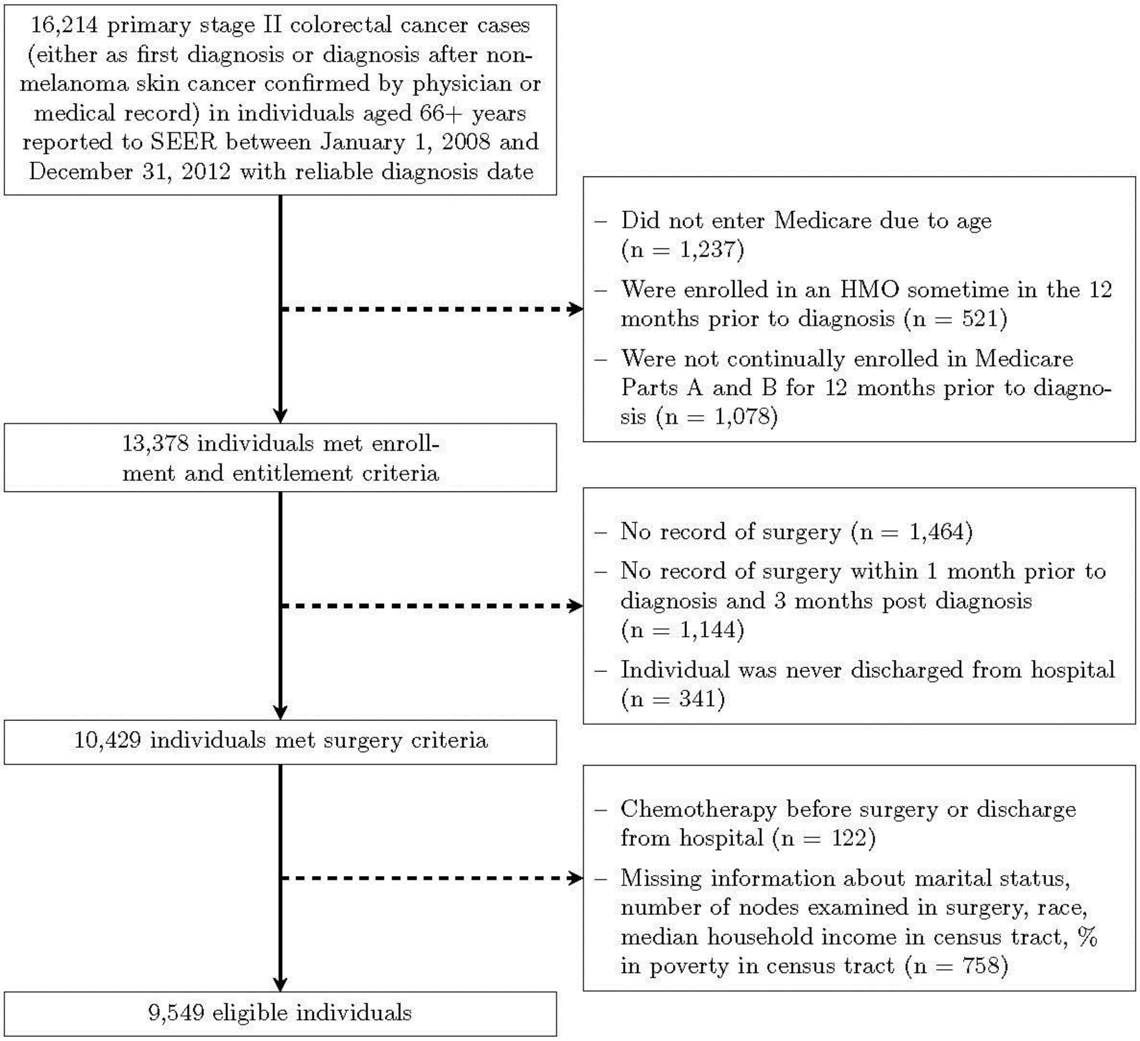

© 2020 Petito LC et al. *JAMA Network Open*.

eFigure 2. Flowchart of Eligibility for a Target Trial of Addition of Erlotinib to Gemcitabine in Individuals With Locally Advanced or Metastatic Pancreatic Cancer, SEER-Medicare 2007-2013

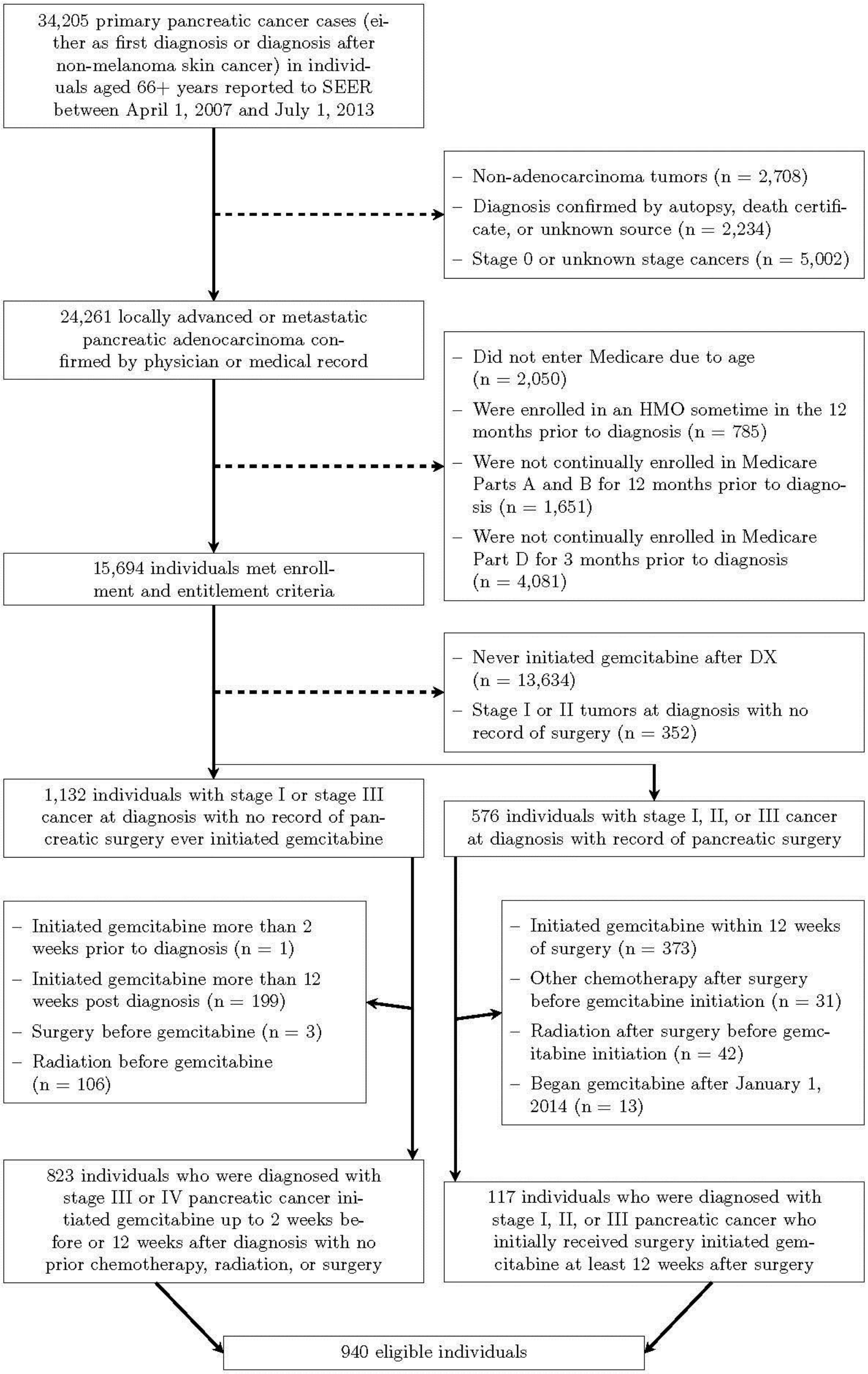

© 2020 Petito LC et al. *JAMA Network Open*.

eFigure 3. Illustration of the Cloning and Censoring Process for the Fluorouracil Target Trial Emulation

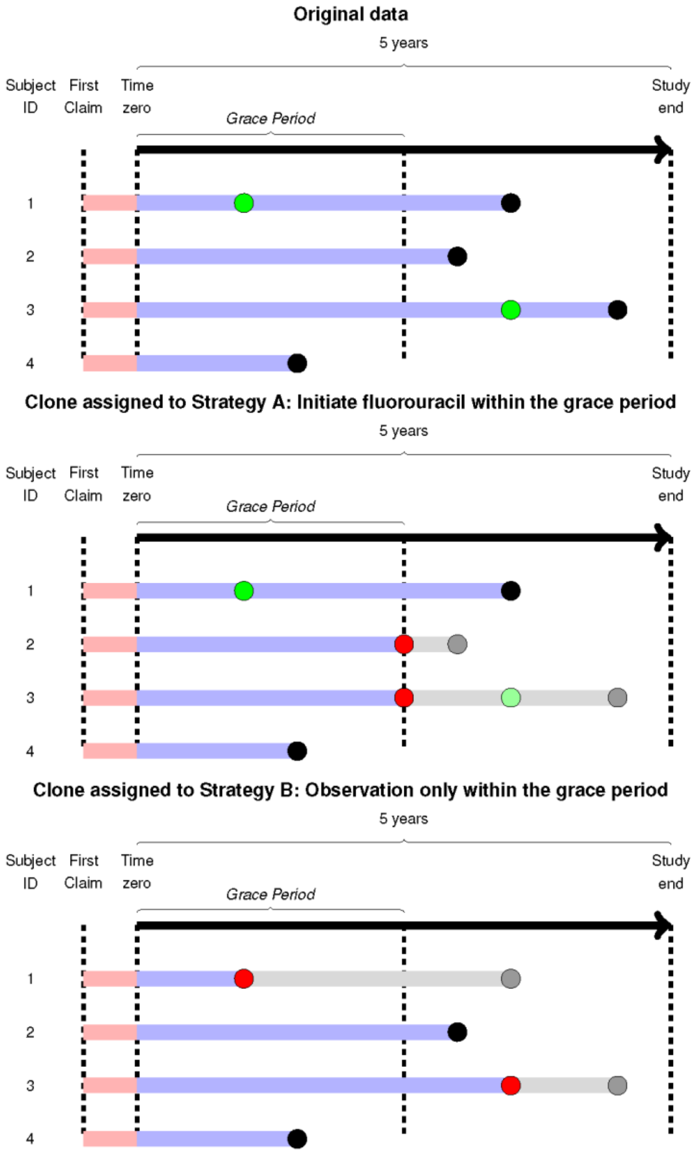

Green circles indicate an instance of fluorouracil. Black circles indicate death or censoring. Red circles indicate artificial censoring. Grey circles indicate death or censoring that occurs after the artificial censoring.

© 2020 Petito LC et al. *JAMA Network Open*.

eAppendix 1. Codes Used to Identify Variables Used in the Analyses

Description	Code source	Codes	Analysis[a]
<u>*Cancer codes*</u>	PDESF file		
Pancreatic cancer	ICD-O-3 recode	21100	E
Non-melanoma skin cancer	ICD-O-3 recode	25020	B
Colorectal cancer	ICD-O-3	C18.0, C18.2, C18.3, C18.4, C18.5, C18.6, C18.7, C18.8, C18.9, C19.9, C20.9, C21.8	F
Adenocarcinoma	ICD-O-3	8140, 8500, 8010, 8560, 8490, 8000, 8260, 8255, 8261, 8263, 8020, 8050, 8141, 8144, 8210, 8211, 8262	E
<u>*Treatment codes*</u>			
Erlotinib	*PDE file only*<br>NDC<br>Brand name<br>Generic	 50242006201, 50242006301, 50242006401<br>Tarceva<br>Erlotinib HCL	E
Gemcitabine	HCPCS/CPT<br>NDC<br>Brand name<br>Generic	J9201<br>00002750101, 00002750201, 00409018101, 00409018201, 00409018501, 00409018601, 00409018701, 00781328275, 00781328379, 16729009203, 16729011711, 25021020810, 47335015340, 47335015440, 55111068607, 55111068725, 63323010213, 63323012550<br>Gemzar<br>Gemcitabine HCL	E
Fluorouracil	HCPCS/CPT<br>NDC	J9190<br>00703301513, 00703301812, 00703301912, 25021021598, 25021021599, 16729027667, 16729027668, 16729027611, 16729027638, 00069016902, 00069017302, 00069017401, 00069017601, 63323011719, 63323011759, 63323011769, 63323011718, 63323011728, 63323011710, 63323011720, 63323011751, 63323011761, 63323011758, 63323011768, 68083026910, 68083027010, 68001026627, 68001026632,	F

© 2020 Petito LC et al. *JAMA Network Open*.

		68001026630, 68001026631, 66758004403, 66758005401, 66758005402	
Other chemotherapy[b]	ICD-9<br><br>Revenue center<br>HCPCS/CPT<br><br><br><br><br><br><br><br><br><br><br><br><br><br><br><br><br><br><br><br><br><br><br><br><br><br><br><br><br><br><br><br><br>NDC	V58.1, V66.2, V67.2, V07.39, 00.10, 17.70, 99.25, 99.28<br>0331, 0332, 0335<br>C1086, C1166, C1167, C1178, C9012, C9110, C9127, C9205, C9207, C9213, C9214, C9215, C9217, C9218, C9235, C9257, C9262, C9414, C9415, C9417, C9418, C9419, C9420, C9421, C9422, C9423, C9424, C9425, C9426, C9427, C9429, C9431, C9432, C9433, C9437, C9440, J0594, J0894, J8510, J8520, J8521, J8530, J8560, J8565, J8600, J8610, J8700, J8705, J8999, J9000, J9001, J9010, J9017, J9020, J9025, J9027, J9033, J9035, J9040, J9041, J9045, J9050, J9055, J9060, J9062, J9065, J9070, J9080, J9090, J9091, J9092, J9093, J9094, J9095, J9096, J9097, J9098, J9100, J9110, J9120, J9130, J9140, J9150, J9151, J9170, J9171, J9178, J9180, J9181, J9182, J9185, J9190, J9200, J9201, J9206, J9207, J9208, J9211, J9230, J9245, J9250, J9260, J9261, J9263, J9264, J9265, J9266, J9268, J9270, J9280, J9290, J9291, J9293, J9300, J9303, J9305, J9307, J9310, J9315, J9320, J9328, J9330, J9340, J9350, J9351, J9355, J9357, J9360, J9370, J9375, J9380, J9390, J9999, Q2017, Q2024, S0087, S0088, S0115, S0116, S0172, S0176, S0178, S0182, C8953, C8954, C8955, G0355, G0357, G0358, G0359, G0360, G0361, G0362, G0370, J7150, Q0083, Q0084, Q0085, S5019, S5020, S9329, S9330, S9331, S9425<br>0519F, 36823, 51720, 61517, 95990, 95991, 99601, 99602, J0640, J0641, 96400-96549	B

© 2020 Petito LC et al. *JAMA Network Open*.

		00004110013, 00004110020, 00004110022, 00004110051, 00004110113, 00004110116, 00004110150, 00004110151, 00005450704, 00005450705, 00005450707, 00005450709, 00005450723, 00005450791, 00015050301, 00015050302, 00015050401, 00015309145, 00054412925, 00054413025, 00054455015, 00054455025, 00054808925, 00054813025, 00054855003, 00054855005, 00054855006, 00054855007, 00054855010, 00054855025, 00081004535, 00085124401, 00085124402, 00085124801, 00085124802, 00085125201, 00085125202, 00085125901, 00085125902, 00173004535, 00173071325, 00182153901, 00182153995, 00364249901, 00364249936, 00378001401, 00378001450, 00378326694, 00536399801, 00536399836, 00555057202, 00555057235, 00555057245, 00555057246, 00555057247, 00555057248, 00555057249, 00555092701, 00555092801, 00555092901, 00555094501, 00603449921, 00677161001, 00781107601, 00781107636, 00904174960, 00904174973, 51079067005, 51079096505, 51285050902, 54569571700, 54868414300, 54868414301, 54868414302, 54868414303, 54868526000, 54868526001, 54868526002, 54868526003, 54868526004, 54868526005, 54868526006, 54868526007, 54868526008, 54868526009, 59911587401, 62701094036, 62701094099, 00703301513, 00703301812, 00703301912, 25021021598, 25021021599, 16729027667,	

© 2020 Petito LC et al. *JAMA Network Open*.

		16729027668, 16729027611, 16729027638, 00069016902, 00069017302, 00069017401, 00069017601, 63323011719, 63323011759, 63323011769, 63323011718, 63323011728, 63323011710, 63323011720, 63323011751, 63323011761, 63323011758, 63323011768, 68083026910, 68083027010, 68001026627, 68001026632, 68001026630, 68001026631, 66758004403, 66758005401, 66758005402	
Radiotherapy	ICD-9<br><br>Revenue center<br>HCPCS/CPT	V58.0, V66.1, V67.1, 92.2x, 92.3x, 92.4, 92.41<br>0330, 0333, 0339<br>G0174, G0251, G0339, G0340, 77401-77499, 77750 - 77899	B
Surgery (pancreatic)			E
Surgery (colorectal)	ICD-9-CM	17.3x, 45.00, 45.03, 45.4x, 45.7x, 45.8x, 46.04, 48.4xx, 48.5xx, 48.6xx	F
Staging tests			
Colonoscopy	HCPCS<br><br>ICD-9	45378, 45380, 45381, 45383, 45384, 45385, G0105, G0121<br>45.23, 45.25	F
Abdominal CT scan	HCPCS<br>ICD-9	74150, 74160, 74170<br>88.01, 88.02	F
Pelvic CT scan	HCPCS	72192, 72193, 72194	F
Emergency room visit	HCPCS	99281, 99282, 99283, 99284, 99285, 99291, 99292	B
Sentinel symptoms[c]			
Intestinal obstruction or perforation	ICD-9	560, 560.8, 560.89, 560.90, 569.83	F
Anemia	ICD-9	280, 280.0, 280.9, 281.9, 285.1, 285.2, 285.22, 285.29, 285.9	F
Abdominal distention	ICD-9	787.3	F
Change in bowel habit	ICD-9	787.99	F
Constipation	ICD-9	564.0, 564.00, 564.01, 564.02, 564.09	F

© 2020 Petito LC et al. *JAMA Network Open*.

Irritable bowel syndrome	ICD-9	564.1	F
Diarrhea	ICD-9	564.5, 787.91	F
Abnormal weight loss	ICD-9	783.2, 783.21, 783.0	F
Asthenia	ICD-9	780.79, 799.3	F

[a] E: erlotinib trial emulation; F: fluorouracil trial emulation; B: both emulations
[b] “Other chemotherapy” excludes revenue center, erlotinib and gemcitabine codes for the erlotinib trial emulation; fluorouracil codes for the fluorouracil trial emulation
[c] Comorbidity identification required one claim only. All available positions were used.

© 2020 Petito LC et al. *JAMA Network Open*.

eAppendix 2. Details of Statistical Analysis

The statistical analysis to estimate the per-protocol effect had three steps: cloning (to avoid immortal time bias), censoring at deviation from protocol (to ensure adherence), and inverse probability weighting (to adjust for selection bias). For simplicity, we will primarily discuss this process in the context of the fluorouracil trial emulation.

Section B.1. Cloning and censoring

The cloning process involved duplicating the original data for each individual and assigning each clone or replicate (two per individual) to either strategy A or B, as visualized in Supplemental Figure 1. We created a new variable to indicate which treatment strategy the replicate was assigned to – this variable is “treated” in the model summaries in Appendices C, D, E, and F.

Replicates were then censored when they deviated from the protocol of the treatment strategy we had assigned them to follow. Each individual’s treatment strategy was completely determined by the end of the grace period, so at most only one replicate from each individual still contributes person-time to the analysis by the end of the grace period.

Supplemental figure 1 illustrates how four types of individuals would be treated in this setting, which we describe in detail here.

Subject 1 initiates fluorouracil (green circle) during the grace period, and then dies or is censored after the grace period ends (black circle). Their complete person-time contributes to the Strategy A clone. However, only their person-time before initiating fluorouracil contributes to the Strategy B clone, resulting in “artificial” censoring (red circle) at the time of fluorouracil initiation.

Subject 2 does not initiate fluorouracil during the grace period, and then dies or is censored after the grace period ends. Their complete person-time contributes to the Strategy B clone. However, only their person-time during the grace period contributes to the Strategy A clone, as they have not initiated fluorouracil by the end of the grace period. The Strategy A clone here is “artificially” censored at the end of the grace period.

Subject 3 initiates fluorouracil after the end of the grace period, and then dies or is censored. Like Subject 2, only their person-time during the grace period contributes to the Strategy A clone, as they have not initiated fluorouracil. Their Strategy B clone only includes the person-time contributed before they initiate fluorouracil – they are “artificially” censored at that time.

Subject 4 dies or is censored during the grace period. In addition to the censoring for administrative or insurance reasons, this censoring includes initiating other chemotherapy for Strategy B. For Strategy A, it also includes initiating other chemotherapy *before*

© 2020 Petito LC et al. *JAMA Network Open*.

initiating fluorouracil. Individuals like Subject 4 contribute their complete person-time to both clones.

Note: For simplicity, we consider censoring due to losing insurance to happen at random, so we do not account for it in our analysis (for example, by using time-varying inverse probability of censoring weights).

Section B.2. Weighting process

For each target trial emulation, we estimated subject-specific time-varying stabilized inverse-probability (IP) weights, which create a pseudopopulation where time-varying prognostic factors are independent of future treatment. To introduce the IP weights, we first have to introduce a bit of notation. A_k is an indicator for use of fluorouracil (or erlotinib) at time *k* (1: ever initiated, 0: never initiated), L_0 is the vector of baseline prognostic factors, and L_k is the vector of time-varying prognostic factors at time *k*. The overbar denotes the history of a variable since start of follow-up. The stabilized IP weights can then be written as:

$$SW_t = \prod_{k=0}^{t} \frac{f(A_k|\bar{A}_{k-1}, L_0)}{f(A_k|\bar{A}_{k-1}, \bar{L}_k)},$$

where

$$f(\,A_k \mid \bar{A}_{k-1}, L_0\,) = \begin{cases} \Pr(A_k = 1 \mid \bar{A}_{k-1}, L_0), & A_k = 1 \\ 1 - \Pr(A_k = 1 \mid \bar{A}_{k-1}, L_0), & A_k = 0. \end{cases}$$

We can similarly define $f(A_k \mid \bar{A}_{k-1}, \bar{L}_k)$.

Fluorouracil in stage II colorectal cancer

To estimate the probabilities in the numerator and denominator, we fit two separate pooled logistic regression model for initiation of fluorouracil in the original, unexpanded study population (n = 9,549). Each model also included a function of time $f(t)$ as restricted cubic splines with knots pre-selected at 3, 16, 30, 44, and 57 months.

The numerator model included baseline covariates: year of diagnosis, sex, race, marital status at diagnosis, region of the US, metropolitan county, median household income in census tract, % households under poverty line in census tract, time between diagnosis and surgery, prolonged hospitalization after surgery, preoperative radiotherapy, cancer type, tumor grade, and comorbidities (anemia, abdominal distention, abnormal weight loss, asthenia, change in bowel movements, constipation, diarrhea, irritable bowel syndrome, # of emergency department visits, colonoscopy, and abdominal or pelvic CT scan). The denominator model included the baseline covariates as well as the most recent measurement of the following time-varying covariates: anemia, abdominal distention,

© 2020 Petito LC et al. *JAMA Network Open*.

abnormal weight loss, asthenia, change in bowel movements, constipation, diarrhea, irritable bowel syndrome, # of emergency department visits, colonoscopy, and abdominal or pelvic CT scan. During months in which a covariate measurement was not available, we carried forward the most recently recorded measurement.

Erlotinib in metastatic pancreatic cancer

To estimate the models in the numerator and denominator, we fit a pooled logistic regression model for initiation of erlotinib in the original, unexpanded study population (n = 940). Each model also included a function of time $f(t)$ as linear and quadratic terms.

The numerator model included baseline covariates: tumor stage, age at diagnosis, and in the year before diagnosis, number of emergency department visits, Charlson Comorbidity index, performance status, cholangitis, and pneumonia. The denominator model included the baseline covariates as well as the most recent measurement of the following time-varying covariates: number of emergency department visits, Charlson Comorbidity index, cholangitis, and pneumonia. During weeks in which a covariate measurement was not available, we carried forward the most recently recorded measurement.

Section B.3. Weighted outcome model

The IP weighted outcome regression is then fit using a pooled logistic regression model:

$$logit(\Pr(Y_{t+1} = 1 \,|Y_t = 0, A, L_0) = \beta_0 + \overrightarrow{\beta_1} f(t) + \beta_2 A + \overrightarrow{\beta_3} f(t) A + \overrightarrow{\beta_4} L_0$$

The predicted values form this IP weighted model are used to compute the cumulative incidence of mortality.

To calculate a single summary (average) hazard ratio as reported in trials, we use the predicted values from the weighted model to simulate the trajectory of each original individual under complete follow-up (10 simulations per individuals were used to reduce simulation uncertainty), as previously described (Toh et al., 2010). That is, we used the estimated probability of death for a random Bernoulli flip to determine if an individual was alive at a given time. The first instance of death was deemed to be end of follow-up. We then fit an unadjusted Cox proportional hazards model in the simulated data, using the predicted time of end of follow-up as the outcome, and treatment assignment (as determined by the end of the grace period) as the sole predictor. The exponentiated coefficient from this model can be interpreted as the average hazard ratio comparing, say, fluorouracil initiators to non-initiators.

95% confidence intervals were generated using a nonparametric bootstrap with 500 resamples. The estimated weights were then truncated at the 99th percentile.

© 2020 Petito LC et al. *JAMA Network Open*.

Section B.4. Implementation

R Code to perform these analyses is available at:

https://github.com/lpetito/SEERMedicareCEAnalysis

In this github repository, we also include a document that describes in great detail how to create the input dataset for these types of analyses.

Additionally, we direct the readers to existing SAS code to implement this type of analysis, the 'Initiators' macro, available at:

https://www.hsph.harvard.edu/causal/software/

A worked example of the analysis of the Coronary Drug Project, an older randomized trial, is available at:

https://github.com/eleanormurray/CausalSurvivalWorkshop_2019

eReference

Toh, S., Hernandez-Diaz, S., Logan, R., Robins, J., and Hernán, M. Estimating absolute risks in the presence of nonadherence: An application to a follow-up study with baseline randomization. *Epidemiology*, 2010; 21(4): 528-539.

© 2020 Petito LC et al. *JAMA Network Open*.

eAppendix 3. Models Used in the Emulation of the Fluorouracil Target Trial

Section C.1. Model coefficients for hazard ratio estimates

Note: In all reported models, t represents the linear term for time, and t*, t**, and t*** represent the estimates for the 1st, 2nd, and 3rd spline basis terms (knots prespecified at 3, 16, 30, 44, and 57 months).

From the unadjusted model (without product term)

	Estimate	Std. Error
Intercept	-4.339399	0.048883
t	-0.051215	0.006478
t*	0.217710	0.046279
t**	-0.511636	0.142585
t***	0.459383	0.209772
treated	-0.000395	0.058004

From the adjusted model (without product term)

	Estimate	Std. Error
Intercept	-4.5559	0.1451
t	-0.0475	0.0066
t*	0.2223	0.0467
t**	-0.5233	0.1436
t***	0.4680	0.2110
treated	0.0183	0.0587
Diagnosed in 2010-2011	0.0095	0.0456
Male	-0.1071	0.0436
Non-Hispanic Black	-0.2090	0.0825
Hispanic/Other	-0.1990	0.0742
Married	-0.3305	0.0437
Region: NE	-0.0292	0.0473
Region: S	0.1213	0.0801
Region: MW	0.0085	0.0629
Urban center	0.0278	0.0570
Median HHI	0.0000	0.0000
% Poverty	0.0068	0.0028
Time between DX and Fluoro: 1-30D	-0.0754	0.0459

© 2020 Petito LC et al. *JAMA Network Open*.

Time between DX and Fluoro: 31-60D	-0.2228	0.0731
Time between DX and Fluoro: 61-90D	0.0122	0.1211
Prolonged post-surgery hospitalization	0.8301	0.0492
Pre-operative radiation	0.2508	0.0575
Rectal cancer	0.1590	0.0948
Both Colon and Rectal cancer	0.2689	0.0848
Grade: poor	-0.1545	0.0489
Anemia (b)	0.1177	0.0499
Abdominal Distension (b)	0.0835	0.0800
Abnormal weight loss (b)	0.2528	0.0509
Asthenia (b)	0.0998	0.0424
Change in bowel movement (b)	0.0152	0.0674
Constipation (b)	0.0623	0.0514
Diarrhea (b)	0.0415	0.0546
Irritable bowel syndrome (b)	-0.1601	0.1286
At least 1 ED visit (b)	0.4024	0.0466
Colonoscopy (b)	-0.4659	0.0458
Abdominal or pelvic CT scan (b)	0.0028	0.0497
Charlson (b)	0.1530	0.0094

From the adjusted *weighted* model (without product term)

	Estimate	Std. Error
Intercept	-4.4866	0.1454
t	-0.0549	0.0065
t*	0.2658	0.0462
t**	-0.6347	0.1420
t***	0.5711	0.2078
treated	-0.0585	0.0550
Diagnosed in 2010-2011	0.0176	0.0458
Male	-0.1145	0.0435
NH Black	-0.1546	0.0802
Hispanic/Other	-0.1724	0.0731
Married	-0.3414	0.0436
Region: NE	-0.0238	0.0473
Region: S	0.0734	0.0812
Region: MW	-0.0047	0.0628

© 2020 Petito LC et al. *JAMA Network Open*.

Urban center	0.0278	0.0570
Median HHI	0.0000	0.0000
% Poverty	0.0063	0.0028
Time between DX and Fluoro: 1-30D	-0.0603	0.0461
Time between DX and Fluoro: 31-60D	-0.2077	0.0730
Time between DX and Fluoro: 61-90D	0.0840	0.1161
Prolonged post-surgery hospitalization	0.8138	0.0494
Pre-operative radiation	0.2692	0.0582
Rectal cancer	0.1119	0.0914
Both Colon and Rectal cancer	0.3133	0.0819
Grade: poor	-0.1403	0.0491
Anemia (b)	0.0889	0.0495
Abdominal Distension (b)	0.1057	0.0779
Abnormal weight loss (b)	0.2433	0.0509
Asthenia (b)	0.1318	0.0425
Change in bowel movement (b)	0.0669	0.0654
Constipation (b)	0.0795	0.0513
Diarrhea (b)	0.0141	0.0549
Irritable bowel syndrome (b)	-0.2062	0.1311
At least 1 ED visit (b)	0.3701	0.0466
Colonoscopy (b)	-0.4135	0.0461
Abdominal or pelvic CT scan (b)	-0.0190	0.0498
Charlson (b)	0.1540	0.0095

© 2020 Petito LC et al. *JAMA Network Open*.

Section C.2. Model coefficients for risk estimates

From the adjusted weighted model with product terms between time and treatment.

	Estimate	Std. Error
Intercept	-4.5598	0.1464
t	-0.0435	0.0070
t*	0.2072	0.0487
t**	-0.4985	0.1487
t***	0.4772	0.2163
treated	0.2100	0.0812
treated x t	-0.1022	0.0232
treated x t*	0.5457	0.1830
treated x t**	-1.1769	0.5660
treated x t***	0.5105	0.8337
Diagnosed in 2010-2011	0.0176	0.0457
Male	-0.1172	0.0434
NH Black	-0.1486	0.0801
Hispanic/Other	-0.1679	0.0729
Married	-0.3370	0.0435
Region: NE	-0.0199	0.0472
Region: S	0.0745	0.0810
Region: MW	0.0027	0.0626
Urban center	0.0310	0.0569
Median HHI	0.0000	0.0000
% Poverty	0.0059	0.0028
Time between DX and Fluoro: 1-30D	-0.0629	0.0460
Time between DX and Fluoro: 31-60D	-0.2127	0.0727
Time between DX and Fluoro: 61-90D	0.0893	0.1158
Prolonged post-surgery hospitalization	0.8122	0.0493
Pre-operative radiation	0.2632	0.0581
Rectal cancer	0.1376	0.0914
Both Colon and Rectal cancer	0.3238	0.0816
Grade: poor	-0.1387	0.0490
Anemia (b)	0.0946	0.0493
Abdominal Distension (b)	0.1131	0.0776

© 2020 Petito LC et al. *JAMA Network Open*.

Abnormal weight loss (b)	0.2462	0.0508
Asthenia (b)	0.1226	0.0425
Change in bowel movement (b)	0.0695	0.0652
Constipation (b)	0.0830	0.0511
Diarrhea (b)	0.0167	0.0547
Irritable bowel syndrome (b)	-0.2115	0.1307
At least 1 ED visit (b)	0.3743	0.0464
Colonoscopy (b)	-0.4142	0.0460
Abdominal or pelvic CT scan (b)	-0.0115	0.0497
Charlson (b)	0.1532	0.0095

© 2020 Petito LC et al. *JAMA Network Open*.

Section C.3. Model coefficients for numerator and denominator of weights

From the model for the numerator of the weights (adjusted for baseline covariates only).

	Estimate	Std. Error
Intercept	-2.8139	0.0920
t	0.0782	0.0042
t*	-0.3552	0.0266
t**	0.8955	0.0791
t***	-0.9103	0.1117
Diagnosed in 2010-2011	0.0633	0.0282
Male	-0.3133	0.0249
NH Black	0.3962	0.0488
Hispanic/Other	0.4919	0.0397
Married	0.4515	0.0259
Region: NE	0.7009	0.0283
Region: S	-0.1204	0.0591
Region: MW	0.5761	0.0351
Urban center	0.3388	0.0306
Median HHI	0.0000	0.0000
% Poverty	-0.0219	0.0019
Time between DX and Fluoro: 1-30D	-0.3379	0.0276
Time between DX and Fluoro: 31-60D	-0.5339	0.0414
Time between DX and Fluoro: 61-90D	-0.5279	0.0751
Prolonged post-surgery hospitalization	-0.2634	0.0483
Pre-operative radiation	-0.8624	0.0599
Rectal cancer	1.3841	0.0392
Both Colon and Rectal cancer	0.5576	0.0438
Grade: poor	-0.3297	0.0283
Anemia (b)	-0.0644	0.0256
Abdominal Distension (b)	0.0272	0.0544
Abnormal weight loss (b)	0.0410	0.0325
Asthenia (b)	-0.4566	0.0278
Change in bowel movement (b)	0.1400	0.0345
Constipation (b)	0.1407	0.0310
Diarrhea (b)	-0.0055	0.0348
Irritable bowel syndrome (b)	-0.3897	0.0813

© 2020 Petito LC et al. *JAMA Network Open*.

At least 1 ED visit (b)	0.0571	0.0257
Colonoscopy (b)	-0.2922	0.0289
Abdominal or pelvic CT scan (b)	0.1973	0.0311
Charlson (b)	-0.1549	0.0080

From the model for the denominator of the weights (adjusted for baseline and time-varying covariates).

	Estimate	Std. Error
Intercept	-2.9602	0.0930
t	0.0872	0.0042
t*	-0.3844	0.0267
t**	0.9628	0.0796
t***	-0.9690	0.1122
Diagnosed in 2010-2011	0.0686	0.0284
Male	-0.3332	0.0250
NH Black	0.4151	0.0490
Hispanic/Other	0.4907	0.0398
Married	0.4562	0.0260
Region: NE	0.6751	0.0284
Region: S	-0.1308	0.0592
Region: MW	0.5637	0.0352
Urban center	0.3544	0.0307
Median HHI	0.0000	0.0000
% Poverty	-0.0222	0.0019
Time between DX and Fluoro: 1-30D	-0.3246	0.0278
Time between DX and Fluoro: 31-60D	-0.5218	0.0416
Time between DX and Fluoro: 61-90D	-0.4905	0.0754
Prolonged post-surgery hospitalization	-0.3158	0.0486
Pre-operative radiation	-0.8819	0.0601
Rectal cancer	1.3701	0.0394
Both Colon and Rectal cancer	0.5422	0.0441
Grade: poor	-0.3205	0.0284
Anemia (b)	-0.0816	0.0260
Abdominal Distension (b)	0.0048	0.0548
Abnormal weight loss (b)	0.0370	0.0327
Asthenia (b)	-0.4961	0.0281
Change in bowel movement (b)	0.1375	0.0346

© 2020 Petito LC et al. *JAMA Network Open*.

Constipation (b)	0.1433	0.0311
Diarrhea (b)	-0.0287	0.0350
Irritable bowel syndrome (b)	-0.4145	0.0817
At least 1 ED visit (b)	0.0383	0.0259
Colonoscopy (b)	-0.2948	0.0290
Abdominal or pelvic CT scan (b)	0.1932	0.0312
Charlson (b)	-0.1648	0.0081
Anemia	0.1332	0.0323
Abdominal Distension	-0.7482	0.2637
Abnormal weight loss	0.2792	0.0814
Asthenia	0.4952	0.0433
Change in bowel movement	-0.7973	0.2286
Constipation	0.0249	0.0922
Diarrhea	0.9004	0.0522
At least 1 ED visit	0.1636	0.0489

© 2020 Petito LC et al. *JAMA Network Open*.

eAppendix 4. Models Used in the Emulation of the Erlotinib Target Trial

Section D.1. Model coefficients for hazard ratio models

First, from the unadjusted model (without product term).

	Estimate	Std. Error
Intercept	-3.6588	0.0787
t	0.0206	0.0070
t^2	-0.0004	0.0001
treated	0.0726	0.0767

Second, from the adjusted model (without product term).

	Estimate	Std. Error
Intercept	-1.7428	5.3358
t	0.0258	0.0070
t^2	-0.0004	0.0001
treated	0.0331	0.0762
Stage IV	1.1042	0.0963
Age	-0.0946	0.1411
Age^2	0.0007	0.0009
ER Visit_b	0.0882	0.0361
Charlson_b	0.0765	0.0331
Cholangitis_b	-0.0805	0.0733
Pneumonia_b	0.1228	0.1119
PerfStat_b	0.2026	0.1230

Third, from the adjusted *weighted* model (without product term).

	Estimate	Std. Error
Intercept	-1.6118	5.3257
t	0.0263	0.0069
t^2	-0.0004	0.0001
treated	0.0479	0.0753
Stage IV	1.0952	0.0958
Age	-0.0972	0.1409
Age^2	0.0007	0.0009
ER Visit_b	0.0884	0.0357
Charlson_b	0.0658	0.0328
Cholangitis_b	-0.0838	0.0730
Pneumonia_b	0.1175	0.1123
PerfStat_b	0.2021	0.1229

© 2020 Petito LC et al. *JAMA Network Open*.

Section D.2. Model coefficients for risk estimates

From the adjusted *weighted* model with product terms between time and treatment.

	Estimate	Std. Error
Intercept	-1.9383	5.3429
t	0.0257	0.0081
t^2	-0.0004	0.0001
treated	0.0175	0.1305
treated*t	-0.0069	0.0160
treated*t^2	0.0004	0.0003
Stage IV	1.0730	0.0964
Age	-0.0873	0.1414
Age^2	0.0007	0.0009
ER Visit_b	0.0892	0.0357
Charlson_b	0.0633	0.0327
Cholangitis_b	-0.0896	0.0731
Pneumonia_b	0.1164	0.1123
PerfStat_b	0.2125	0.1231

Section D.3. Model coefficients for numerator and denominator of weights

From the model for the numerator of the weights (adjusted for baseline covariates only).

	Estimate	Std. Error
Intercept	-71.0660	54.3223
t	0.3295	0.0743
t^2	-0.0035	0.0013
treated_b	7.1252	1.3004
Stage IV	0.8562	0.6807
Age	1.6136	1.4584
Age^2	-0.0110	0.0098
ER Visit_b	-0.1548	0.2890
Charlson_b	-0.1365	0.2400
Cholangitis_b	0.3159	0.5265
Pneumonia_b	-0.3275	1.0530
PerfStat_b	0.2621	1.0338

From the model for the denominator of the weights (adjusted for baseline and time-varying covariates).

	Estimate	Std. Error
Intercept	-70.8648	54.2720
t	0.3288	0.0743
t^2	-0.0035	0.0013
treated_b	7.1318	1.3009
Stage IV	0.8616	0.6874

© 2020 Petito LC et al. *JAMA Network Open*.

Age	1.6065	1.4572
Age^2	-0.0109	0.0098
ER Visit_b	-0.1574	0.2898
Charlson_b	-0.1494	0.2474
Cholangitis_b	0.3224	0.5297
Pneumonia_b	-0.3431	1.0545
PerfStat_b	0.2606	1.0326
ER Visit	0.0303	0.3214
Charlson	0.0377	0.2456
Cholangitis	-0.2000	1.9267
Pneumonia	-0.1070	2.1838

© 2020 Petito LC et al. *JAMA Network Open*.

eAppendix 5. Sensitivity Analyses for the Fluorouracil Target Trial Emulation

Section E.1. Time modeled as a restricted cubic spline with 3 knots

	HR	95% CI
Unadjusted	1.09	0.97 to 1.21
Adjusted for baseline variables	1.08	
Adjusted for baseline and time-varying variables	1.07	

HR: hazard ratio; CI: confidence interval

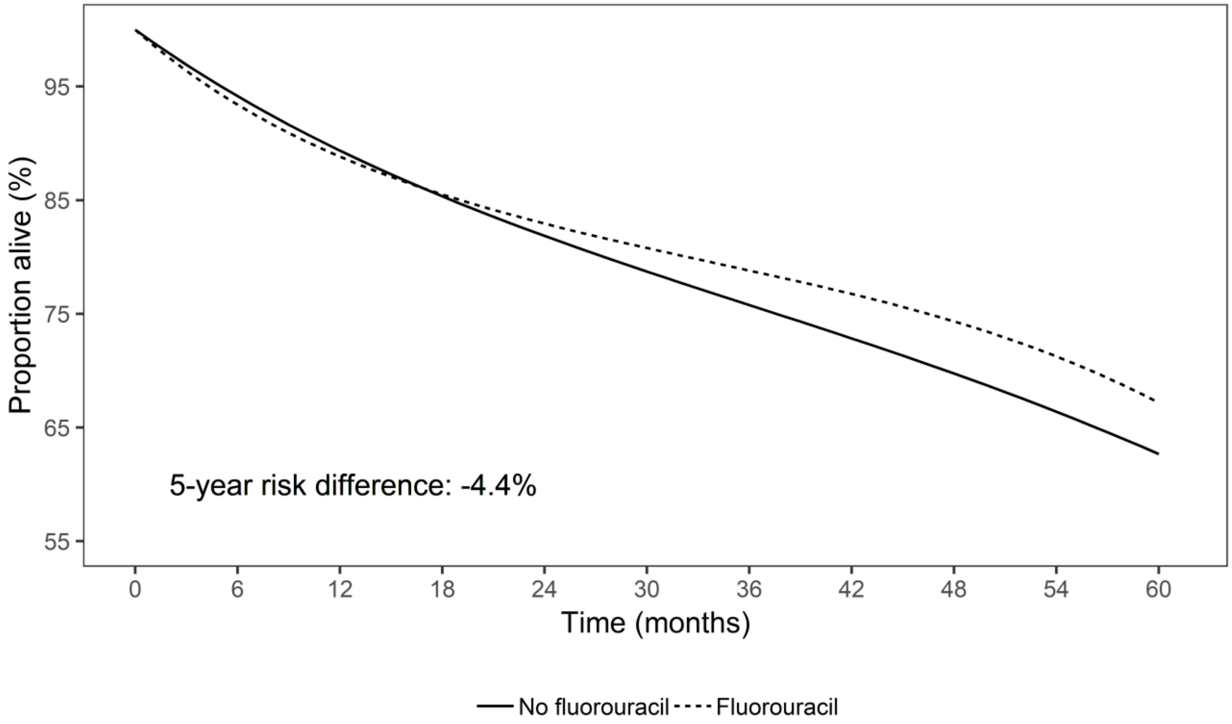

© 2020 Petito LC et al. *JAMA Network Open*.

Section E.2. Time modeled as a restricted cubic spline with 4 knots

	HR	95% CI
Unadjusted model	1.02	0.91 to 1.14
Adjusted for baseline variables	1.03	
Adjusted for baseline and time-varying variables	1.02	

HR: hazard ratio; CI: confidence interval

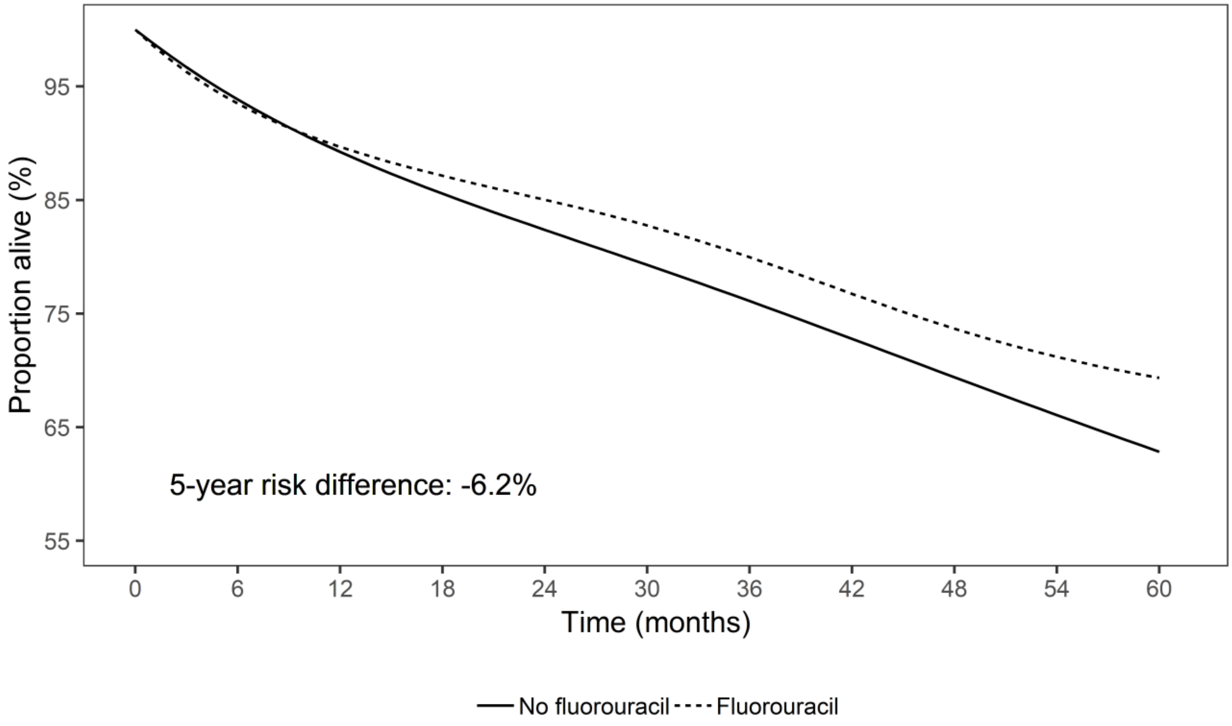

© 2020 Petito LC et al. *JAMA Network Open*.

Section E.3. Grace period duration: 1 month

	HR	95% CI
Unadjusted model	1.07	0.94 to 1.21
Adjusted for baseline variables		
Adjusted for baseline and time-varying variables		

HR: hazard ratio; CI: confidence interval

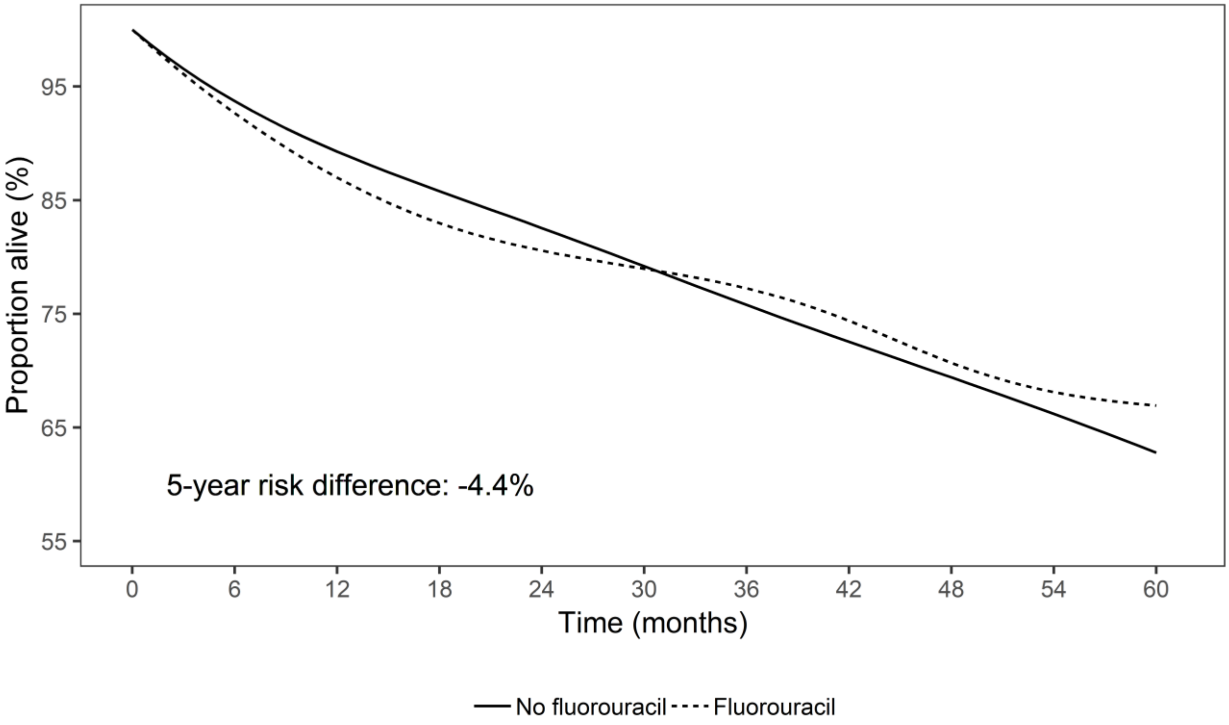

© 2020 Petito LC et al. *JAMA Network Open*.

Section E.4. Grace period duration: 6 months

	HR	95% CI
Unadjusted model	0.96	0.87 to 1.06
Adjusted for baseline variables	0.98	
Adjusted for baseline and time-varying variables	0.95	

HR: hazard ratio; CI: confidence interval

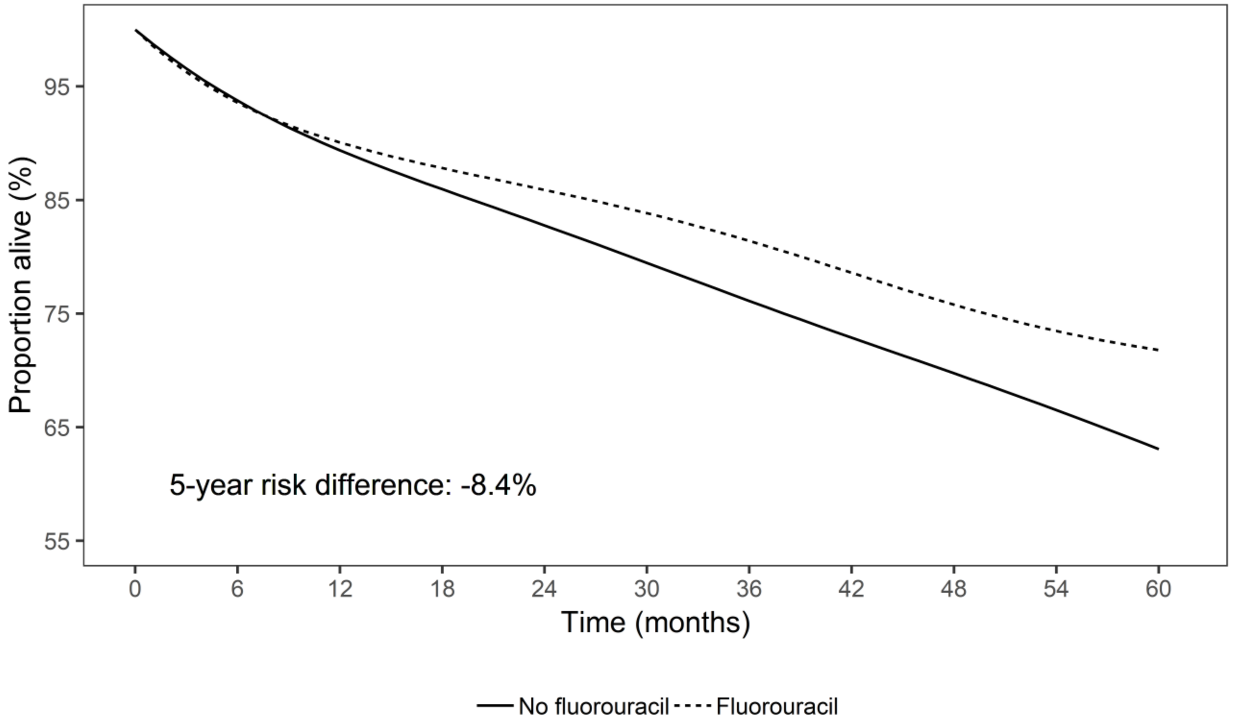

© 2020 Petito LC et al. *JAMA Network Open*.

eAppendix 6. Sensitivity Analyses for the Erlotinib Target Trial Emulation

Section F.1. Time modeled as linear

	HR	95% CI
Unadjusted	1.03	0.89 to 1.20
Adjusted for baseline variables	1.00	
Adjusted for baseline and time-varying variables	1.03	

HR: hazard ratio; CI: Confidence Interval

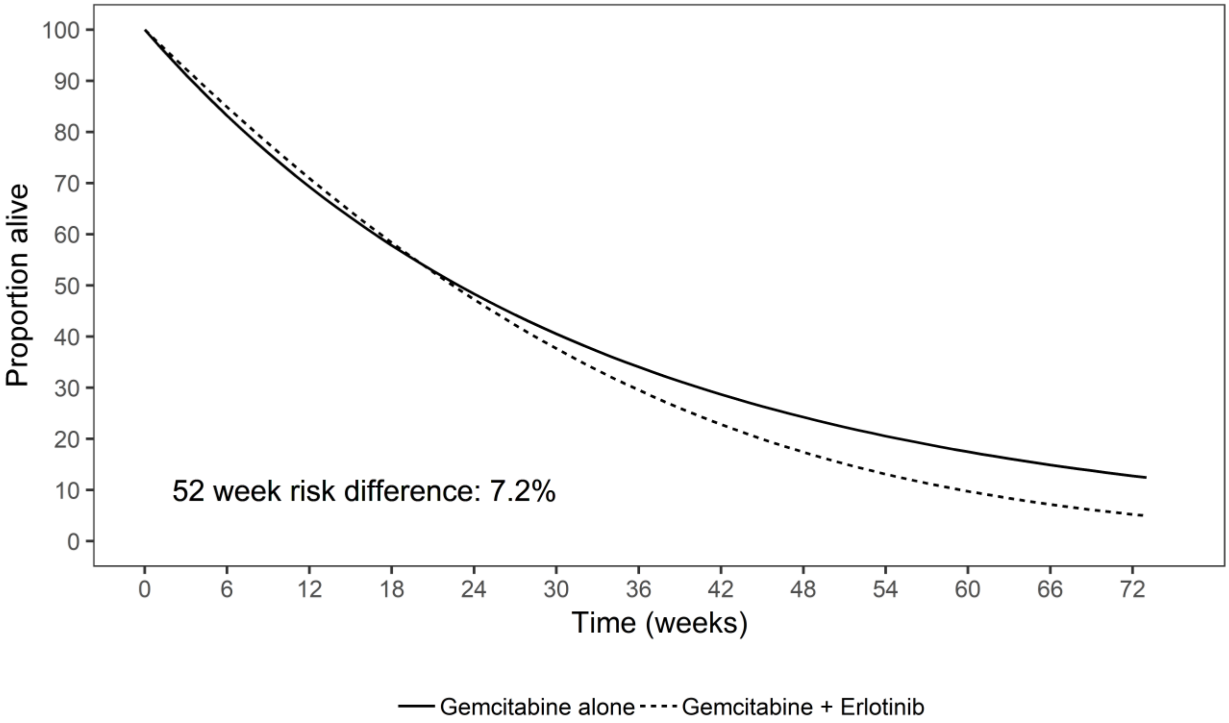

© 2020 Petito LC et al. *JAMA Network Open*.

Section F.2. Time modeled as a restricted cubic spline with 3 knots

	HR	95% CI
Unadjusted	1.08	0.92 to 1.25
Adjusted for baseline variables	1.03	
Adjusted for baseline and time-varying variables	1.04	

HR: hazard ratio; CI: Confidence Interval

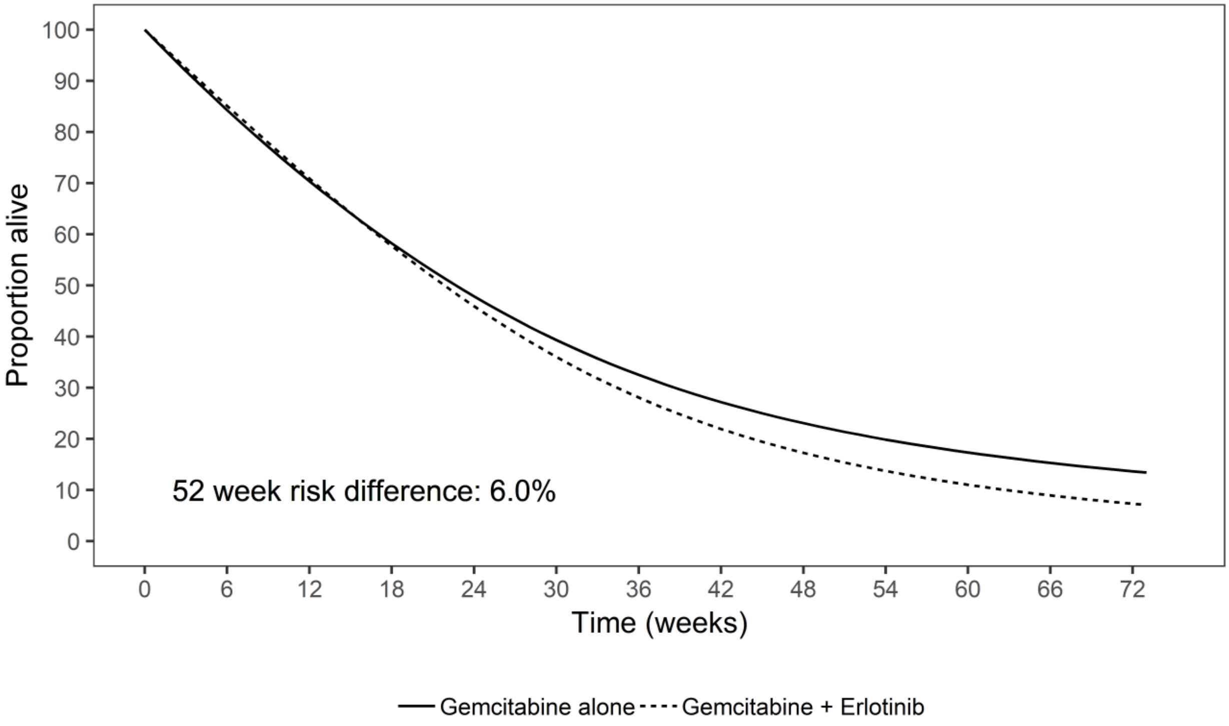

© 2020 Petito LC et al. *JAMA Network Open*.

Section F.3. Grace period duration: 6 weeks

	HR	95% CI
Unadjusted	1.09	0.91 to 1.30
Adjusted for baseline variables	1.04	
Adjusted for baseline and time-varying variables	1.05	

HR: hazard ratio; CI: Confidence Interval

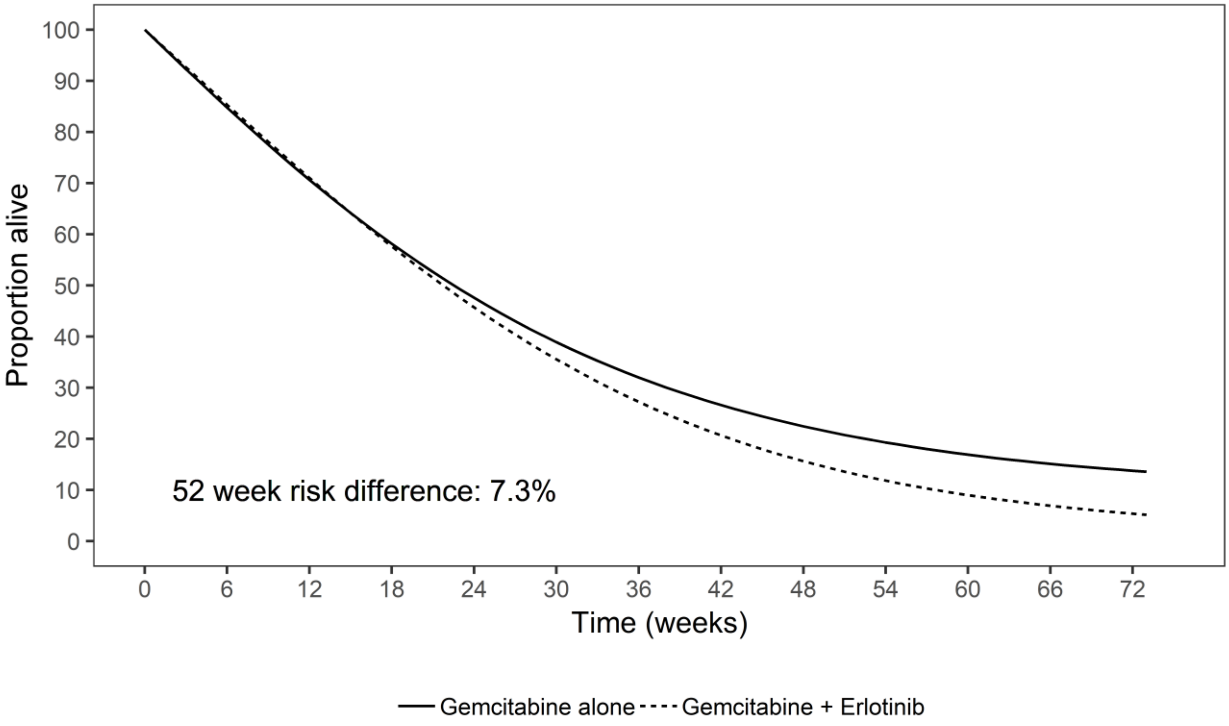

© 2020 Petito LC et al. *JAMA Network Open*.

Section F.4. Grace period duration: 24 weeks

	HR	95% CI
Unadjusted	1.06	0.93 to 1.20
Adjusted for baseline variables	1.02	
Adjusted for baseline and time-varying variables	1.02	

HR: hazard ratio; CI: Confidence Interval

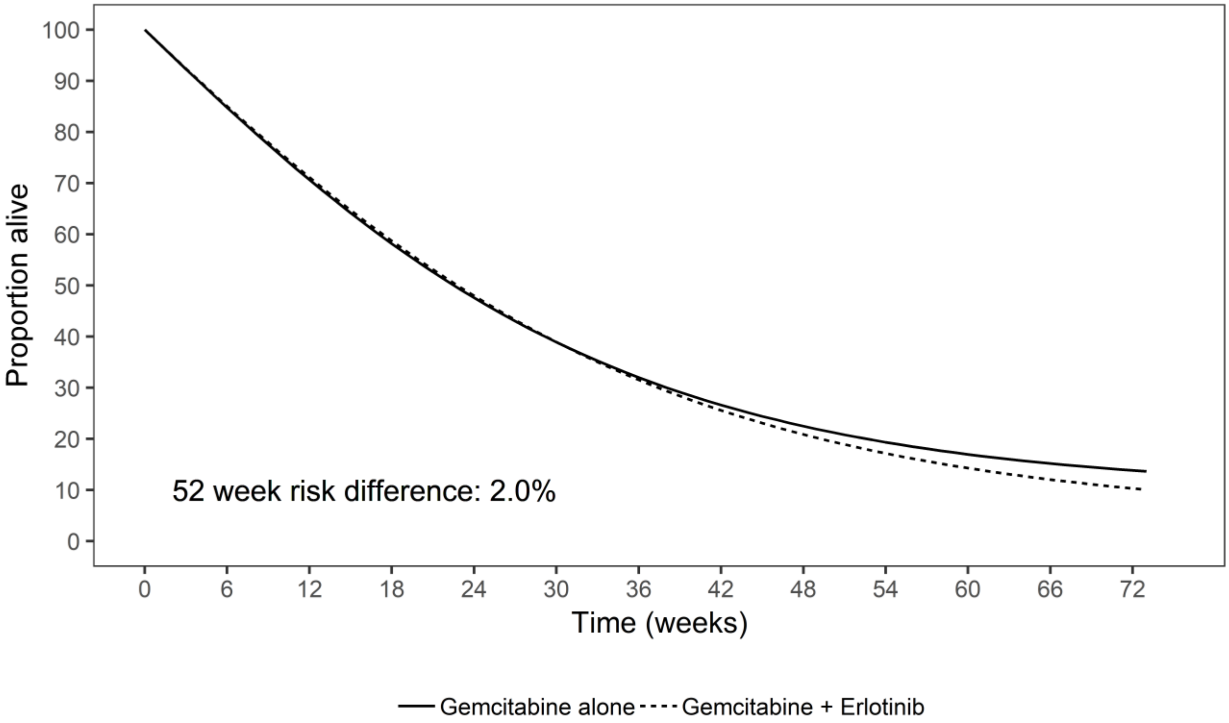

© 2020 Petito LC et al. *JAMA Network Open*.
